# Identification and validation of a dysregulated TME-related gene signature for predicting prognosis, and immunological properties in bladder cancer

**DOI:** 10.3389/fimmu.2023.1213947

**Published:** 2023-10-27

**Authors:** Chong Shen, Wang Chai, Jingwen Han, Zhe Zhang, Xuejing Liu, Shaobo Yang, Yinlei Wang, Donghuai Wang, Fangxin Wan, Zhenqian Fan, Hailong Hu

**Affiliations:** ^1^ Department of Urology, The Second Hospital of Tianjin Medical University, Tianjin, China; ^2^ Tianjin Key Laboratory of Urology, Tianjin Institute of Urology, Tianjin, China; ^3^ Obstetrics and Gynecology, Haidian Maternal & Child Health Hospital, Beijing, China; ^4^ Department of Gastrointestinal Surgery, The Second Hospital of Tianjin Medical University, Tianjin, China; ^5^ Department of Endocrinology, The Second Hospital of Tianjin Medical University, Tianjin, China

**Keywords:** bladder cancer, tumor microenvironment (TME), prognosis model, immunotherapy, drug sensitivity

## Abstract

**Background:**

During tumor growth, tumor cells interact with their tumor microenvironment (TME) resulting in the development of heterogeneous tumors that promote tumor occurrence and progression. Recently, there has been extensive attention on TME as a possible therapeutic target for cancers. However, an accurate TME-related prediction model is urgently needed to aid in the assessment of patients’ prognoses and therapeutic value, and to assist in clinical decision-making. As such, this study aimed to develop and validate a new prognostic model based on TME-associated genes for BC patients.

**Methods:**

Transcriptome data and clinical information for BC patients were extracted from The Cancer Genome Atlas (TCGA) database. Gene Expression Omnibus (GEO) and IMvigor210 databases, along with the MSigDB, were utilized to identify genes associated with TMEs (TMRGs). A consensus clustering approach was used to identify molecular clusters associated with TMEs. LASSO Cox regression analysis was conducted to establish a prognostic TMRG-related signature, with verifications being successfully conducted internally and externally. Gene ontology (GO), KEGG, and single-sample gene set enrichment analyses (ssGSEA) were performed to investigate the underlying mechanisms. The potential response to ICB therapy was estimated using the Tumor Immune Dysfunction and Exclusion (TIDE) algorithm and Immunophenoscore (IPS). Additionally, it was found that the expression level of certain genes in the model was significantly correlated with objective responses to anti-PD-1 or anti-PD-L1 treatment in the IMvigor210, GSE111636, GSE176307, or Truce01 (registration number NCT04730219) cohorts. Finally, real-time PCR validation was performed on 10 paired tissue samples, and *in vitro* cytological experiments were also conducted on BC cell lines.

**Results:**

In BC patients, 133 genes differentially expressed that were associated with prognosis in TME. Consensus clustering analysis revealed three distinct clinicopathological characteristics and survival outcomes. A novel prognostic model based on nine TMRGs (including C3orf62, DPYSL2, GZMA, SERPINB3, RHCG, PTPRR, STMN3, TMPRSS4, COMP) was identified, and a TMEscore for OS prediction was constructed, with its reliable predictive performance in BC patients being validated. MultiCox analysis showed that the risk score was an independent prognostic factor. A nomogram was developed to facilitate the clinical viability of TMEscore. Based on GO and KEGG enrichment analyses, biological processes related to ECM and collagen binding were significantly enriched among high-risk individuals. In addition, the low-risk group, characterized by a higher number of infiltrating CD8+ T cells and a lower burden of tumor mutations, demonstrated a longer survival time. Our study also found that TMEscore correlated with drug susceptibility, immune cell infiltration, and the prediction of immunotherapy efficacy. Lastly, we identified SERPINB3 as significantly promoting BC cells migration and invasion through differential expression validation and *in vitro* phenotypic experiments.

**Conclusion:**

Our study developed a prognostic model based on nine TMRGs that accurately and stably predicted survival, guiding individual treatment for patients with BC, and providing new therapeutic strategies for the disease.

## 1 Introduction

Globally, bladder cancer (BC) ranks as the 10th most common tumor, with an increasing incidence and mortality rate, resulting in a heavy social burden (1). According to GLOBOCAN estimates, the number of new cases and deaths worldwide in 2020 came to 573,278 and 212,536 respectively (2). According to newly diagnosed BC, 70% of all bladder cancers are non-muscle invasive (NMIBC). The best option for bladder tumors is transurethral resection (TURBT). Tumor recurrence or progression can occur in up to 45% and 6% to 17% of patients after TURBT, respectively (3). Likewise, while radical cystectomy and neoadjuvant platinum-based chemotherapy have been used clinically in patients with muscle-invasive bladder cancer (MIBC), poor quality of life and intolerance and insensitivity to chemotherapy in some patients are the greatest challenges in this field (4). In recent years, BC can be targeted with targeted therapy in the future, but only specific types of patients benefit (5). Moreover, immunotherapy has been a significant clinical advance, and immune checkpoint inhibitors (ICIs) therapeutics are displacing previous treatment regimens as first- and second-line therapies for BC patients (6). It is unfortunate that immunotherapy doesn’t show a high rate of response, and these drugs are relatively expensive. Therefore, researching specific prognostic biomarkers that can be used to categorize patients with different characteristics, and identifying new therapeutic strategies, has important clinical application value.

The tumor microenvironment (TME), as the “soil” of tumor cells, consists of various factors, including immune cells, endothelial cells, fibroblasts, extracellular matrix (ECM), and secreted growth factors (7). Growing evidence suggests a crucial role for TME in tumorigenesis, progression, metastasis, and response to therapies (8). The acquisition and maintenance of cancer differentially depend on contributions from TME components. Recently, TME has attracted broad clinical interest as a therapeutic target in cancer (9). Thus, intensively investigate complexity of TME is essential for understanding the mechanisms of cancer progression and boosting the predictive power of immunotherapy. However, to date, whether TME-related gene signatures are a novel prognostic model in BC remains unclear.

From public databases, we retrieved mRNA expression profiles and corresponding clinical data for BC patients to assess their prognosis. Next, an extensive molecular clustering analysis was performed, and a novel TME-related gene signature was constructed to provide a prognostic model. Next, we confirmed the mRNA expression of interested model-associated genes from 10 paired BC tissues collected by us. Besides, we further investigated their impact on infiltrating immune cells, functional enrichment pathways, and therapeutic response with BC patients. Meanwhile, the modeled genes were also tested in different immunotherapy cohorts for potential biomarkers for immunotherapy efficacy (i.e., IMvigor210, GSE111636, GSE176307, or our Truce01). Of note, we carried out a series of cellular function experiments following SERPINB3 knockdown. Our results demonstrated that TMEscore is a robust potential prognostic biomarker and therapeutic target. This study would contribute to providing new insights for developing viable treatment strategies for BC.

## 2 Materials and methods

### 2.1 Study design

**Figure 1:**
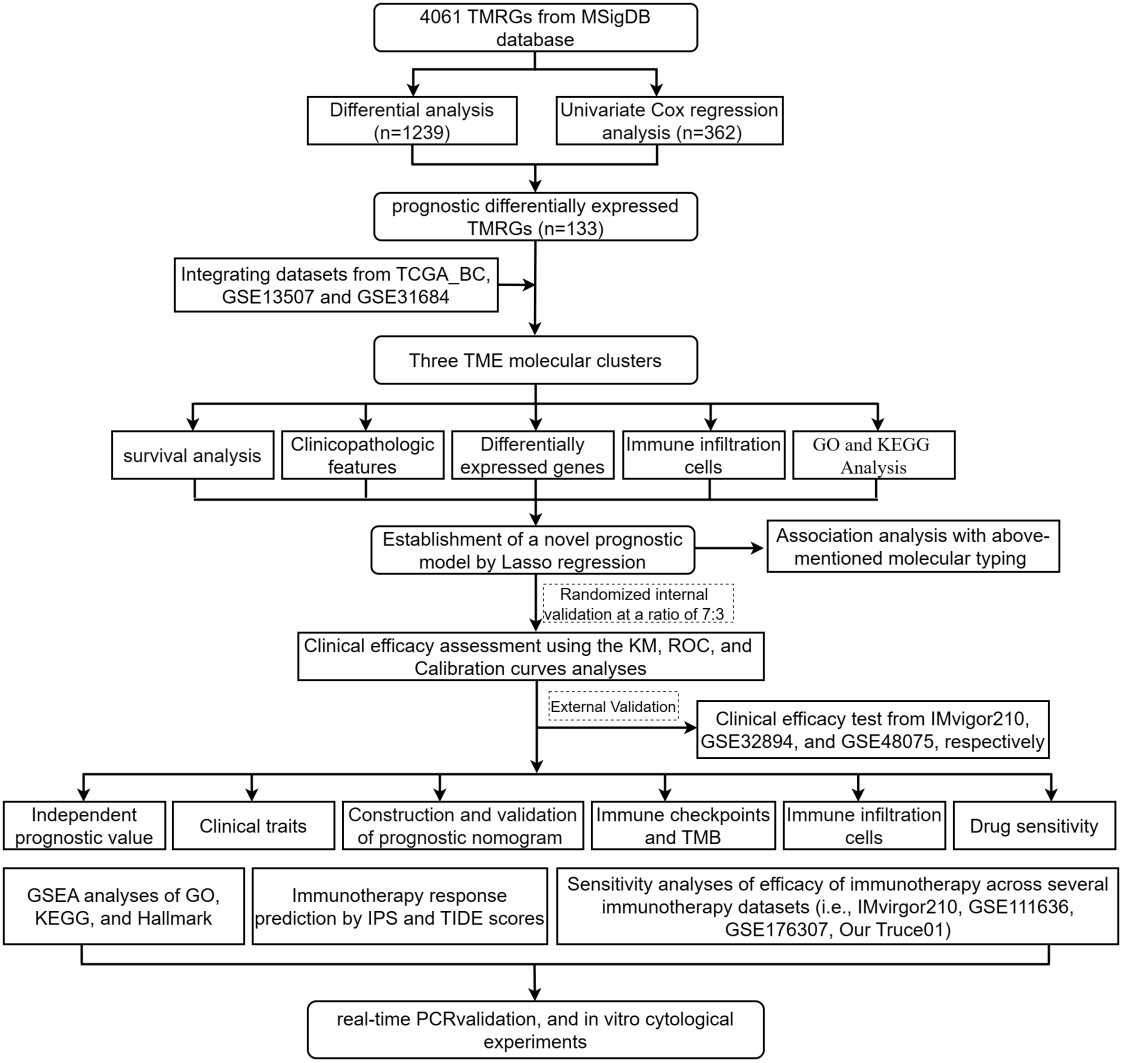
Flow chart of our study.

The workflow of our study is depicted in **Figure 1**.

### 2.2 Data collection and preprocessing

Transcriptome profiling data and corresponding clinical data for BC were retrieved from The Cancer Genome Atlas (TCGA) database (https://portal.gdc.cancer.gov), Gene Expression Omnibus (GEO) database (https://www.ncbi.nlm.nih.gov/gds), and the IMvigor210 database. Patients lacking survival information were excluded. Totally, TCGA-BC and 2 eligible GEO cohorts (GSE13507, GSE31684) were gathered for this study. In the Affy software package, raw microarray data from Affymetrix was processed using the RMA algorithm for background adjustment and quantile normalization. We directly downloaded the normalized matrix files from Illumina with the raw data. For the TCGA dataset, the transcriptome data (FPKM values) were downloaded from the Genomic Data Commons (GDC, https://portal.gdc.cancer.gov/) using the TCGA biolinks bioconductor package (10). Then FPKM values were converted to transcripts per kilobase million (TPM) using the “limma” R package for analysis. This dataset includes 414 tumor and 19 tumor-adjacent samples and is used to compare the expression levels of TME-related genes in tumors and normal tissues. For GEO datasets, probe-level annotations from the AffyMetrix platform are converted to gene symbols (11). Batch effects between TCGA and GEO datasets were corrected using the “Combat” algorithm of the SVA dataset. Somatic mutations and copy number variations (CNVs) from UCSC Xena (https://gdc.xenahubs.net/). Data were analyzed with R Bioconductor packages.

### 2.3 Identification of prognostic differentially expressed TME-related genes

A total of 4061 TMRGs were identified from the MSigDB database (MSigDB, https://www.gsea-msigdb.org/gsea/msigdb/search.jsp). To screen differentially expressed TMRGs (DETMRGs) with the threshold of false discovery rate (FDR)< 0.05 and |log2 fold-change (FC)| > 1 between tumor and adjacent tissue from the TCGA dataset, the “limma” R package and Wilcoxon test were utilized. Meanwhile, univariate Cox regression (uniCox) analysis was conducted to screen TME-related prognostic genes with the threshold of p-value< 0.01. After screening, view Venn diagrams and heatmaps with the Venn diagram analysis website and heatmap R package. Furthermore, gene mutation frequencies were obtained using the “maftools” R package. CNVs for each gene were analyzed and visualized using the Rcircos package (12).

The protein-protein interaction (PPI) network predicted using DETMRG (13) was constructed using the STRING database (https://string-db.org/) between proteins with a confidence limit of 0.8 for interactions. Networks were analyzed and visualized using Cytoscape software version 3.9.1.

### 2.4 Identifying TME-related molecular clusters with consensus clustering analysis

Transcriptome profile data and corresponding clinical data from TCGA-BC, GSE13507 and GSE31684 datasets were merged for analysis. To identify molecular clusters associated with the TME, 1000 iterations of consensus clustering analysis were performed using the CancerSubtypes R package to ensure the robustness of the classification (14). The relationship of TME-related molecular clusters with clinicopathologic characteristics and survival outcomes was explored with the R packages “survival”, “limma”, “ggplot2”, and “pheatmap”.

### 2.5 Identification of differentially expressed genes and functional enrichment analysis

To identify differentially expressed genes (DEGs) in distinct TME clusters, the “limma” package was used with criteria of |log2 FC| > 0.585 and FDR< 0.05. (which implements an empirical Bayesian approach to estimate gene-expression changes using moderated t-tests). Expression data of DEGs from different TME clusters were normalized in BC samples and crossover genes were extracted. To explore potential mechanisms among these DEGs, Gene Oncology (GO) and Kyoto Encyclopedia of Genes and Genomes (KEGG) functional enrichment analysis was carried out with the “clusterProfiler” package (15). In addition, single-sample gene set enrichment analysis (ssGSEA) was used to quantify the enriched fractions of 23 tumor-infiltrating immune cells (16). Differences in biological processes between molecular patterns were investigated using the Gene Set Variation Analysis (GSVA) R package.

### 2.6 Establishment and verification of a novel prognostic model for BC

UniCox analysis was applied to investigate prognosis-related DEGs. A total of 224 survival-related genes were extracted for further analysis. The consensus clustering algorithm was used for defining the number of gene clusters and their stability. In our study, the combined data from TCGA-BC, GSE13507 and GSE31684 datasets (664 BC cases) were randomly separated into a training cohort (468 BC cases) and testing cohort (196 BC cases) in a 7:3 ratio. Meanwhile, 348 BC samples from the Vigor210 database were used as an external validating cohort. Subsequently, we performed LASSO regression with 10-fold cross-validation to narrow the prognosis-related DEGs applying the R package “glmnet”, and multivariate Cox regression analysis in the training cohort to establish a novel prognostic model for BC. Finally, the TMEscore of each patient was calculated based on the following model formula:


$$\text{risk score}=\text{Sicorrespomding Coefficient}\left(\text{mRNAi}\right)\times \text{Expression}\left(\text{mRNAi}\right)$$

Kaplan–Meier (K-M) survival analysis, time-dependent receiver operating characteristic (ROC) curve analysis and principal component analysis (PCA) were performed to validate the performance of this prognostic model in the training cohort, testing cohort, entire and external validating cohort, respectively. The distribution of risks across all cohorts was visualized using the R package “pheatmap”. The association of TME gene signature with clinical variables was discussed, and categorical analyses were also performed to investigate whether TMEscore still had predictive reliability for multiple clinicopathological features based on different subgroups. MultiCox analysis was applied to determine whether the risk score was an independent prognostic predictor. Interestingly, subgroup survival analyses were performed for older and younger patients, males and females, T-stage I-II and III-IV, low-grade and high-grade patients. In addition, the model of 18 glycolysis-related genes was validated in three independent research groups. (i.e., IMvigor210, GSE48075, and GSE32894). Afterward, a nomogram through the “rms” R package was constructed for BC patients with TMEscore and clinical characteristics, particularly about 1-, 3-, and 5-year overall survival (OS). The clinical reliability and predictive value of the established nomogram were assessed by calibration plots and ROC, respectively.

### 2.7 Correlation analyses of the TME-related model with immune subtypes, immune checkpoint, immune infiltrating cells, tumor mutation burden, tumor immune dysfunction and exclusion algorithm and immunophenoscore

We first compared the risk score between the four different immunophenotyping with sample size greater than 3, including C1 (wound healing), C2 (INF-r dominance), C3 (inflammation), and C4 (lymphocyte depletion), from the previous study of David et al. (17). To explore the efficacy of treatment response, immune checkpoint genes were compared between high- and low-risk groups. Following, to evaluate the immune infiltration of BC, we used seven immune infiltration algorithms (TIMER, CIBERSORT, CIBERSORT-abs, QUANTISEQ, MCPCOUNTER, XCELL, and EPIC) to calculate the proportion of different immune cells and reveal the immune infiltration function under different strategies. Using differences in risk scores between the two groups were analyzed by Wilcox rank sum test; the results were then visualized as heatmaps using the “Pheatmap” R package. The correlation between risk score and immune infiltrating cells was analyzed by Spearman rank correlation analysis. In addition, 29-point immune function/infiltration score values were evaluated for high-risk and low-risk groups using the single-sample gene set enrichment analysis (ssGSEA) algorithm. TMB was assessed as previously described (18). Furthermore, in order to distinguish the mutation spectrum of BC patients between the two risk groups, we used the “maftools” package (19) to obtain the mutation annotation format (MAF) files of the TCGA database. TMB, TIDE, and IPS scores are all immune checkpoint blockade responses (ICB; anti-PD1 or anti-CTLA4, etc.) that help quantify tumor immunogenicity and characterize the intertumoral immune landscape. Consequently, this model can be used to predict the response of BC patients to immunotherapy.

### 2.8 Drug sensitivity analysis

The immunophenotype score (IPS) was calculated to predict BC patients’ immune responses between the different risk groups. Next, the CellMiner database (https://discover.nci.nih.gov/cellminer/home.do) was applied to reveal whether the nine TMRGs could predict the sensitivity of anticancer drugs. Spearman’s correlation analysis was used to determine the correlation between risk gene expression levels and drug sensitivity.

### 2.9 The correlation between TME-associated model and the efficacy of BC immunotherapy

Gene expression profiles and clinical information were obtained from three independent cohorts (GSE111636, GSE176307, and IMvigor210) and one of our sequence data sets (term_id, TRUCE-01; registration number, NCT04730219; registration time: July 11 2020) and predicted values of gene expression models in response to immunotherapy. In our TRUCE01 study, we also performed comparisons of differences between two groups before and after treatment using a paired Wilcoxon-test for these genes. The results were divided into respond and non-respond, and the statistical difference was set at p<0.05.

### 2.10 RNA extraction, quantitative real-time PCR, cell counting Kit−8 (CCK−8) and transwell assays *in vitro*.

Total RNA was evoked from 10 matched BC tumors and adjacent tissues using the eznam tm Hp Total RNA (OMEGA) kit. Converted to cDNA using the RevertAid First Strand cDNA Synthesis Kit (Thermo Fisher Scientific, Rockford, IL, USA). The relative expression of COMP and SERPINB3 mRNA was detected by QRT-PCR. The COMP primer sequences were: forward, 5’-CGAGTCCGCTGTATCAACACC-3’; reverse, 5’-TCCGTGCAAACCTGCTTGT-3’. SERPINB3 primer: forward, 5’-CGCGGTCTCGTGCTATCTG-3’; reverse, 5’-ATCCGAATCCTACTACAGCGG-3’. The final results were analyzed using the 2^-ΔΔCT^ method.

Cell proliferation was assessed using a CCK-8 experiment. In the CCK-8 assay, 2x10^3^ T24 or 253J-BV cells were seeded into 96-well plates at a density of cells per well. At specified time points (0, 24, 48, 72, and 96 hours), 10 µl of CCK-8 solution (Beyotime Institute of Biotechnology) was added to each well, followed by incubation at 37˚C for an additional 3 hours in the absence of light. The absorbance at a wavelength of 450 nm was then measured using a microplate reader.

5 × 10^4^ BC cells were cultured for cell migration and invasion assays using 200 µl serum-free medium in the upper chamber (0.8 µm; Corning) and 700 µl complete medium in the lower chamber (for invasion assay) or without (for migration assay) Matrigel (Corning), following incubation for 48 h, fixed and treated with 4% Paraformaldehyde (Sigma) and 0.1% crystal violet (Solarbio) were stained, and cell migration and invasion were quantified using an Olympus microscope (Olympus).

### 2.11 Statistical analysis

All analyses were completed by using R programming language (version 4.1.2) and its relevant packages. Wilcox’s test was employed to compare the variables of the two groups. Chi-square tests were utilized to investigate the relationship between risk groups and clinicopathological features. Spearman’s correlation test was used to assess the correlation between groups. The Kaplan-Meier curve was employed to assess the survival data. The R package time was utilized to conduct the ROC analysis. Additionally, both univariate and multivariate Cox regression analyses were conducted to evaluate independent prognostic factors. A two-sided P< 0.05 was considered statistically significant. Furthermore, p-value summaries were as follows: ****, P < 0.0001; ***, 0.0001 < P < 0.001; **, 0.001 < P < 0.01; *, 0.01 < P ≤ 0.05; ns, P > 0.05.

## 3 Results

### 3.1 Screening and Genetic Mutation Landscape of Prognostic DETMRGs

We first identified the expression levels of the 4061 TMRGs in normal and tumor samples based on the TCGA-BC cohort. Subsequently, 1239 DE-TMRGs (**Supplementary Table S1**) and 362 prognostic TMRGs (**Supplementary Table S2**) were found. 133 intersect prognostic DE-TMRGs were extracted and visualized with heatmap (**Supplementary Figures S1A–C**, **Supplementary Table S3**). A PPI network was established to discover de-TMRG interactions affecting prognosis, which indicated that VEGFA, SPP1, and TIMP1 were the three main core genes (**Supplementary Figure S1D**). As depicted in **Supplementary Figure S1E**, 255 of 412 (61.89%) samples in the TCGA-BC cohort presented genetic mutations. The results suggested that AHNAK, FBN2, FBN1, NAV3, and HSPG2 were the top 5 most common mutated genes, and missense mutations were the most frequent types. Then, we explored CNV mutational incidence, and **Supplementary Figure S1F** lists the top 20 genes in the gain and loss-CNV groups, respectively. The Circus plot shows the chromosomal distribution of the prognostic de-tmrg, suggesting that CNVs may play a regulatory role in tmrg expression. Taken together, these results suggest a potential prognostic role of DE-TMRG in BC tumor development and progression.

### 3.2 Identification of three TME-related molecular clusters

**Figure 2:**
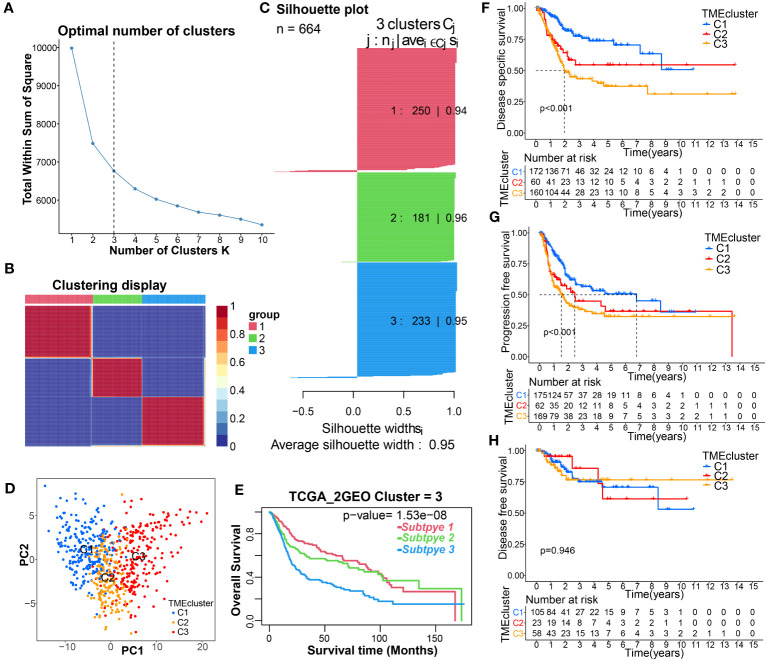
Identification of TME-related molecular clusters using consensus clustering. **(A–C)** Consensus matrix heatmap, and Silhouette plot defining that 3 was the appropriate value of k clusters. **(D)** PCA showing obvious difference in transcriptomes between the three subgroups. **(E–H)** K-M curve for the OS, DSS, PFS, and DFS in patients with different clusters.

**Figure 3:**
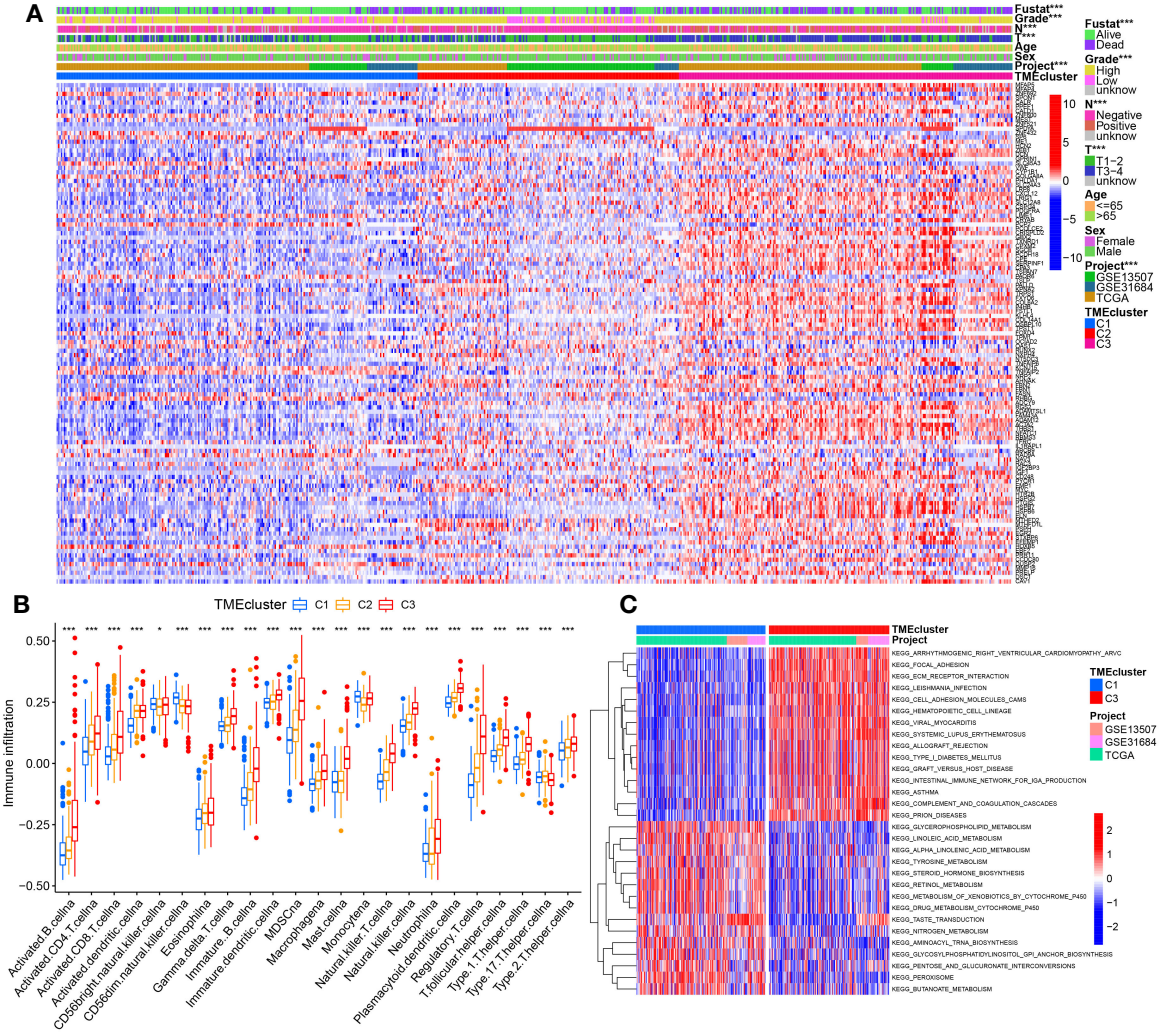
The correlation between molecular clusters and clinicopathological characteristic, immune infiltration levels, or pathway enrichment. **(A)** The heatmap of these differentially prognostic TMGs with correlation of the molecular typing with clinical features at the top by chi-square test. **(B)** Abundance differences of 23 immune infiltrating cell types between different molecular typing. **(C)** Top30 significantly enriched KEGG pathways between C3 vs C1 by GSVA algorithm. *p< 0.05, ***p< 0.001.

An integrative datasets from TCGA-BC, GSE13507 and GSE31684, including 664 BC patients, were further analyzed to investigate the relationship between TME-related genes expression and tumorigenesis, or BC patient outcomes. Complete clinical characteristics of these patients are listed in **Supplementary Table S4**. A sum of 115 prognostic DE-TMRGs were extracted. To classify BC patients according to the expression levels of these genes, we performed an unsupervised analysis. Our results showed that with an optimal clustering variable of 3, BC samples were divided into three clusters – viz, cluster 1 (C1, n = 250), cluster 2 (C2, n = 181) and cluster 3 (C3, n = 233) (**Figures 2A–C**). PCA analysis also confirmed a good distribution between groups (**Figure 2D**). The KM curves showed significant differences in OS (**Figure 2E**), disease-specific survival (DSS, **Figure 2F**), progression-free survival (PFS, **Figure 2G**) among the three subtypes. Patients in C3 had the worst outcome. Despite this, DFS did not differ significantly among the three subgroups (**Figure 2H**). Furthermore, the expression levels of genes in C3 are significantly upregulated and correlated with T-stage, N-stage, grade, and survival status compared with C1 and C2 (**Figure 3A**).

Results from ssGSEA revealed striking differences in the accumulation of most immune cells between the three clusters, including activated B cells, activated CD4 T cells, immature B cells, myeloid suppressor cells (MDSCs), macrophages, hypertrophic cells, natural killer cells, regulatory T cells (Tregs), follicular T helper cells, and type 2 T helper cells were significantly enriched in C3 (**Figure 3B**). The differential results of GSVA accumulation analysis of KEGG pathways based on C3 and C1 dimensions indicated that C3 was mainly enriched in metastasis-related pathways, e.g., C1 was enriched in metabolic pathways (peroxisome, fatty acid metabolism, glycerophospholipid metabolism, linoleic acid metabolism) that were significantly enriched (**Figure 3C**, **Supplementary Table S5**). Additionally, further analysis by the R software package ‘GSVA’ identified that 68, and 74 significantly different KEGG pathways were present in C2 vs. C1, and C3 vs. C2, respectively (**Supplementary Table S5**). C2 is enriched in cell cycle and DNA repair processes such as homologous recombination, DNA replication, spliceosome, nucleotide excision repair, mismatch repair, etc. (**Supplementary Figures S2A, B**). The 24 terms were the intersection of significantly enriched pathways in C2 vs. C1, C3 vs. C2, and C3 vs. C1 (**Supplementary Figure S2C**, **Supplementary Table S5**).

### 3.3 Identification of Gene Subtypes Based on DEGs from molecular typing

**Figure 4:**
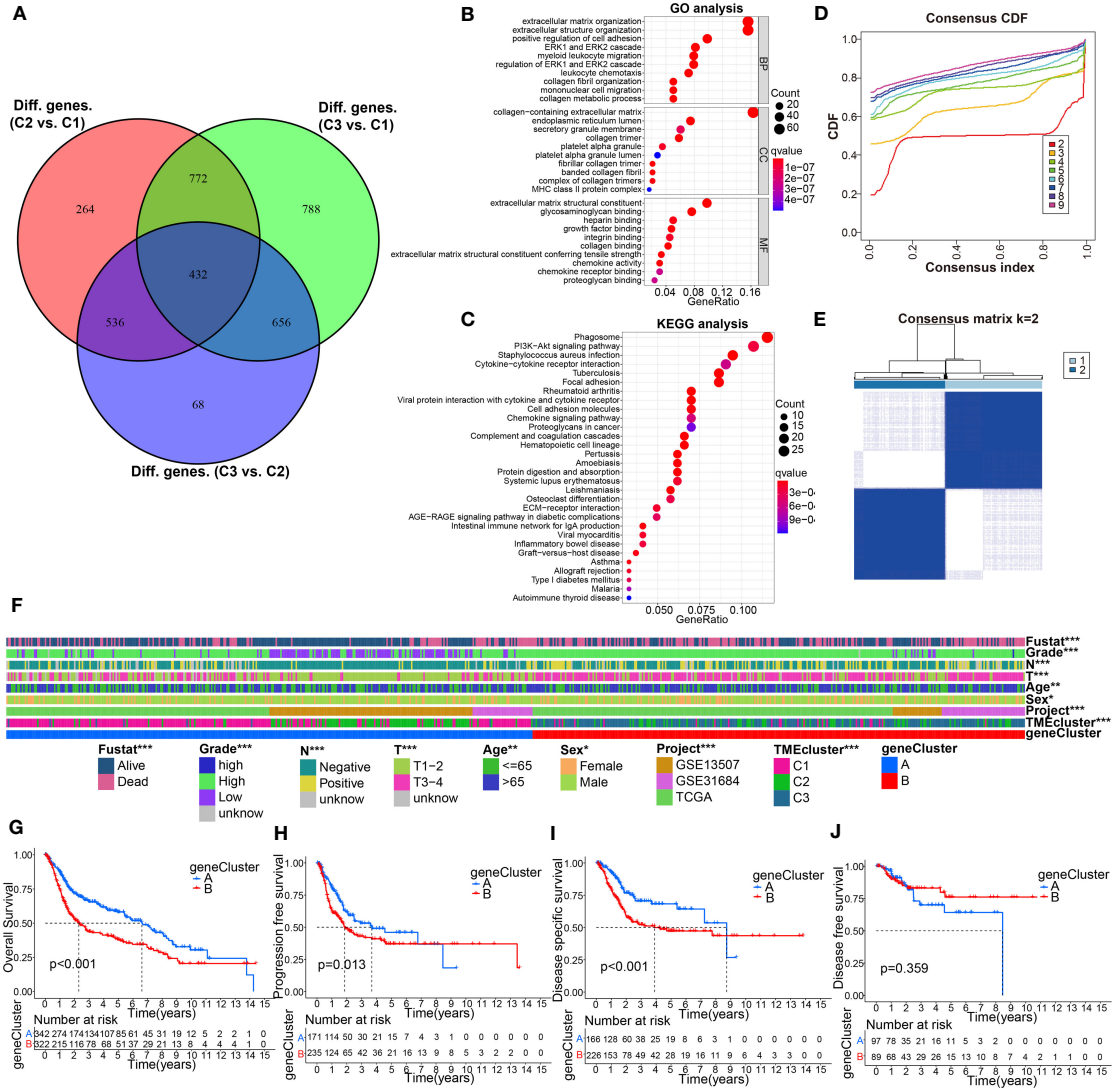
Identification of gene subtypes based on DEGs from comparison of the above molecular classification. **(A)** 432 TME phenotype-related intersect differential genes from C3 vs C1, C3 vs C2, and C2 vs C1, was shown in Venn diagram. **(B, C)** GO and KEGG enrichment analyses for these 432 intersection differential genes. **(D, E)** Genotyping was performed using these 432 genes. **(F)** Correlation analysis of gene signature with clinicopathological characteristics using Chi-square test. **(G–J)** KM curve analysis for OS, PFS, DSS, and DFS of the gene clusters. *p< 0.05, **p< 0.01, ***p< 0.001.

To further investigate the biological behavior of each TME cluster, we identified 432 TME-associated DEGs (**Figure 4A**). We then performed GO and KEGG signaling pathway enrichment analysis on these DEGs (**Supplementary Table S6**), and the results showed that these genes were associated with ECM organization and extracellular structure organization (**Figure 4B**). KEGG analysis also revealed enrichment of PI3K-AKT signaling pathway and ECM receptor interaction (**Figure 4C**), confirming that ECM may play a key role in regulating BC development. In addition, we performed uniCox analysis and identified 224 prognostic-related grades (**Supplementary Table S7**).

To further investigate the underlying mechanism, patients were divided into two gene clusters (clusters A and B) based on the difference in prognosis using consensus clustering analysis (**Figures 4D, E**). We found that the two gene clusters were significantly related to distinct clinicopathological features (**Figure 4F**). Interestingly, K-M curves suggested a remarkable difference of OS, PFS, and DSS between the different subtypes, and BC patients in cluster B had the shortest survival time (**Figures 4G–J**).

### 3.4 Construction and verification of a novel TME gene signature based on DEGs, clinical correlation analysis.

**Figure 5:**
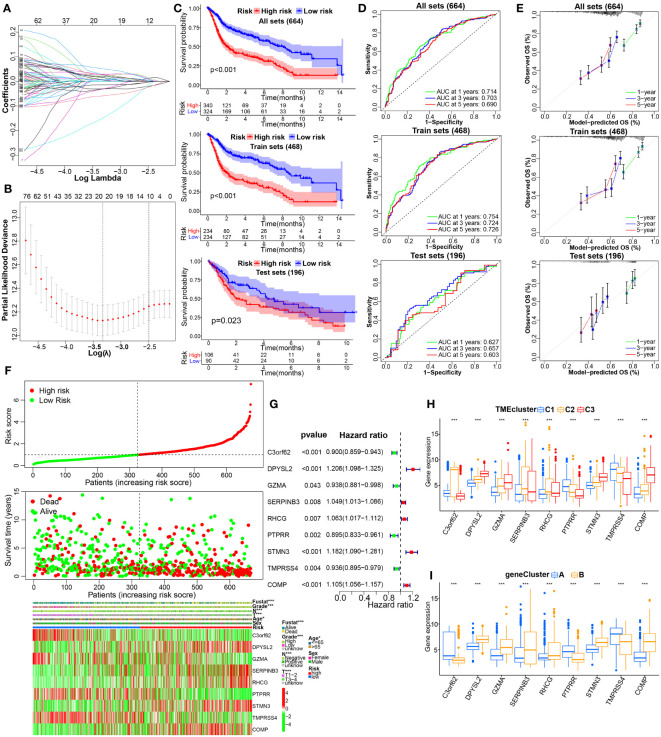
Construction and internal validation of TME-related prognostic signature in BC. **(A, B)** LASSO regression analysis with minimal lambda value. **(C)** The KM survival analysis showing the difference in OS between the high- and low-risk groups in the integrated, training, testing cohorts. **(D, E)** Time-dependent ROC, and calibration curves at 1, 3 and 5 years. **(F)** The distribution of risk score, survival status, and the modeled genes expression heatmap of BC patients with different risk scores in the whole cohort. **(G)** Forest plot of TME‐associated prognostic modeled genes based on univariate Cox regression analysis. **(H, I)** The difference in the expression of TME model genes in the molecular clusters or genotyping depicted above. *p< 0.05, ***p< 0.001.

In order to establish a novel prognostic model for BC, based on the abovementioned results of uniCox analysis, we next performed LASSO and multiCox analysis in the training cohort (**Figures 5A, B**). Ultimately, 9 model genes were screened out, and the calculation results of TMEscore were as follows: Risk score = (-0.099662222260127) * C3orf62 + (0.106039015724471) * DPYSL2 + (-0.25538986534307) * GZMA + (0.085706581025753) * SERPINB3+ (0.0782409611222662) * RHCG + (-0.081618821104433) * PTPRR + (0.102457210158663) * STMN3+ (-0.1019226467793) * TMPRSS4+ (0.0712031406107373) * COMP. According to the median risk score of the training group, patients were divided into high-risk and low-risk groups, and divided into training group and verification group. KM survival analysis showed that the OS of high-risk patients was significantly lower than that of low-risk patients in the training cohort (p< 0.001), testing cohort (p = 0.023), whole cohort (p< 0.001) (**Figures 5C**); the AUC values of 1-, 3-, and 5-year prognosis in the training cohort were 0.754, 0.724, and 0.726, severally, similar to the results for the test cohort and the entire cohort (**Figure 5D**). In addition, calibration curves predicting 1-, 3-, and 5-year OS in training, testing, and full cohorts are shown in **Figure 5E**. Analogously, the risk map assessed showed that as the risk assessment increased, survival time decreased and mortality increased (**Figure 5F**). "Moreover, univariate Cox regression analysis confirmed the prognostic significance of these nine model genes, and then the findings were presented in a." forest plot (**Figure 5G**). To further investigate the link between modelled gene expression and the aforementioned molecular classifications, we plotted the distribution of modelled gene expression among different molecular typing using a boxplot (**Figures 5H–I**). Next, to examine the relationship between the model and clinical characteristics, we used chi-square tests or Wilcox nonparametric tests on pooled datasets to compare risk scores for different clinical characteristics. As determined in **Supplementary Figure S3**, patients with >65 age, high grade, stage_T3-4, positive lymphatic invasion and survival status dead, compared to ≤65 age, low grade, stage_T1-2, negative lymphatic invasion, and survival status alive had higher risk scores or risk ratios, respectively.

**Figure 6:**
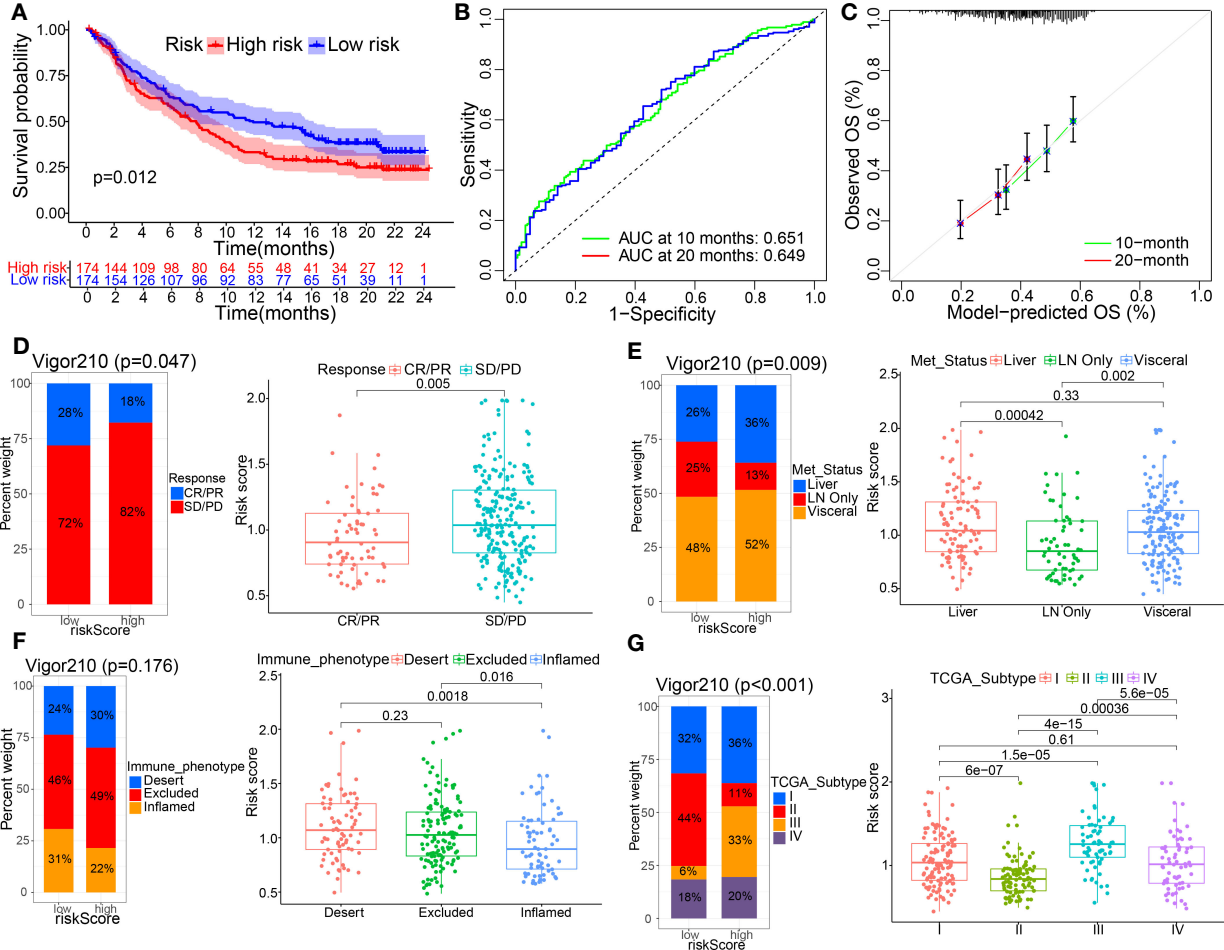
External verification of TME-related prognostic signature was made on IMvigor210 datasets. **(A–C)** KM curves and their 1-, 3-, 5-year ROC and calibration curves were displayed. **(D–G)** The correlations between the model and clinicopathological parameters, including immunotherapy efficacy, metastasis status, immunophenotyping, and TCGA_subtype, using Chi-square test and Wilcox nonparametric test.

To confirm the reliability of existing prognostic prediction models, we evaluated the predictive ability of the models on several external validation datasets. KM, ROC and calibration curve analyses were also performed for the model based on IMvigor210, GSE32894, and GSE48075 datasets, and showed good performance of the predictive models, see **Figures 6A–C**, **Supplementary Figures 4A–C**, **4H–J**. Of note, we also observed significant correlations between THUMPD1 expression and immunotherapy efficacy, status of distant metastasis, immunophenotyping, TCGA_subtype, and previous polecular typing through Chi-square or Wilcox nonparametric tests (**Figures 6D–G**, **Supplementary Figures 4D–G**). Altogether, these results manifest the superior performance of this new prognostic model in predicting the prognosis of BC patients.

### 3.5 Independent prognosis analysis, nomogram construction, and functional enrichment analysis

**Figure 7:**
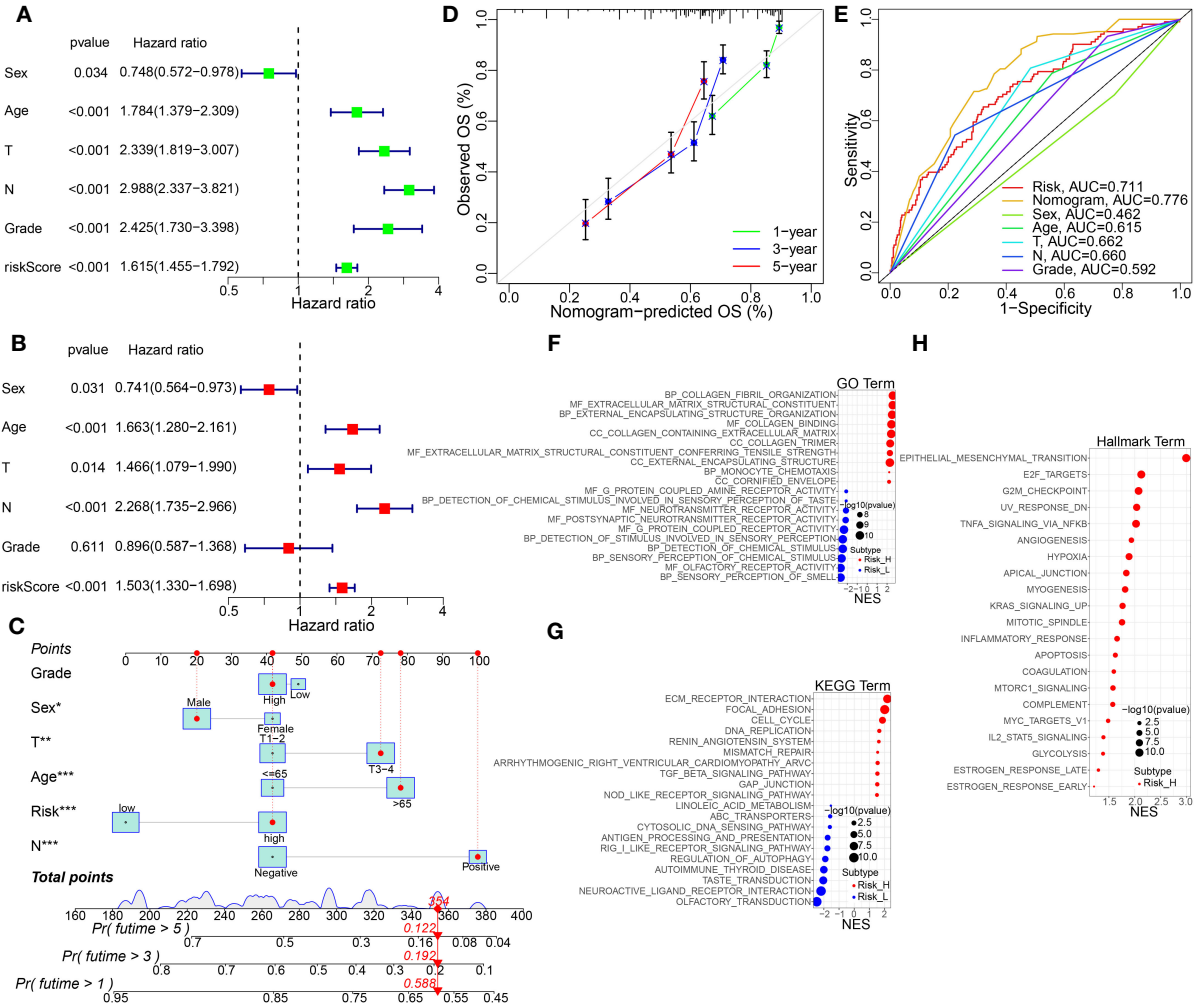
The independent prognosis analysis, nomogram construction and validation, and KEGG pathway enrichment analysis by GSVA method. **(A, B)** Univariate and multivariate Cox regression analysis of the risk score and other clinical features in the integrated cohort. **(C)** Nomogram for predicting the 1-, 3-, and 5-year OS of BC patients in the whole cohort. **(D)** Calibration curve for the OS nomogram model in BC. A dashed diagonal line represents the ideal nomogram. **(E)** ROC curve of the nomogram model combined with common clinic traits. **(F–H)** GO, KEGG, and Hallmarker pathway analysis using GSVA algorithm in the high- vs. low-risk groups. *p<0.05, **p<0.01, ***p< 0.001.

Furthermore, we performed uniCox and multiCox analyses to evaluate the independent prognostic value of TMEscore in BC patients. The results showed that age, T stage, N stage and risk score were independent adverse prognostic factors for BC patients. (**Figures 7A, B**). Additionally, subgroup survival analysis showed that high-risk patients have lower survival rates than low-risk patients. (**Supplementary Figure S5**).

To further advance the usage of our prognostic model, a risk score was used to construct a nomogram (**Figure 7C**), incorporating multiple pathological features clinically. Calibration curves verified the high agreement of actual and predicted OS rates in BC patients between 1, 3, and 5 years (**Figure 7D**). In addition, as shown in **Figure 7E**, ROC demonstrated that the predictive ability of the road map for BC prognosis is excellent. Functional enrichment analysis of GO, KEGG and Hallmarker pathways showed that biological processes and pathways related to ECM, hypoxia, and collagen binding were significantly enriched in the high-risk group (**Figures 7F–H**).

### 3.6 Association of the prognostic model with immune infiltration cells and immune checkpoints

**Figure 8:**
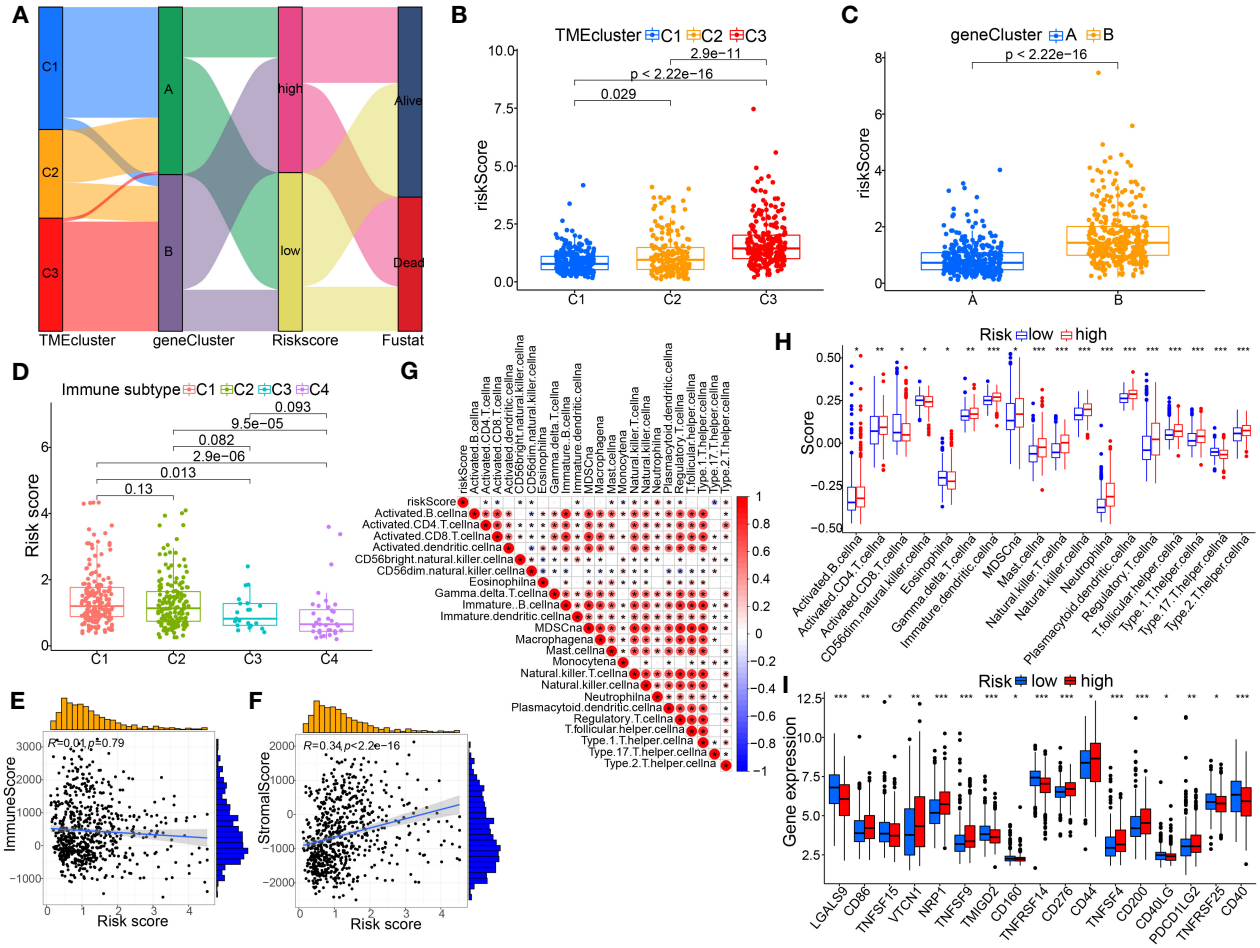
Association of the TME-based signature with molecular clusters, genotyping or other immunological features. **(A)** Sankey diagram showing the relationship between the TME clusters, gene clusters, risk score, and survival status. **(B)** Differences in risk score among the TME-related molecular clusters. **(C)** Differences in risk score between the two gene subtypes. **(D)** Comparison of riskscores between different immune subtypes. **(E, F)** Spearman correlation analysis between the risk score and immune or stromal score. **(G, H)** Association between risk score and immune infiltrating cells by spearman correlation test and Wilcox nonparametric test, respectively. **(I)** The differentially expressed immune checkpoint-related genes between the two risk groups. p< 0.05 *; p< 0.01 **; p< 0.001 ***.

Sankey diagram displayed the connection among the TMEclusters, gene clusters, riskscore groups, and survival status (**Figure 8A**). We observed significant differences in the risk scores of the TME clusters and gene clusters, C3 and gene cluster B had the highest risk score, and C1 and gene cluster A had the lowest (**Figures 8B, C**).

The present study investigated the association between the signature in BC tissue and four immune subtypes with sample size greater than 3, including C1 (wound healing, n=173), C2 (INF-r dominance, n=164), C3 (inflammation, n=21), as well as C4 (lymphocyte depletion, n=36) (**Figure 8D**). In BC tissues, the riskscore of the model was highest in C1 and lowest in C4. Meanwhile, we also analyzed the correlation between immunological and stromal scores calculated using this model and those assessed by the R package (**Figures 8E, F**). The results illustrated the significantly positive correlation between riskscore and stromal scores; whereas, no remarkable correlation for riskscore with immune scores. Furthermore, the results of ssGSEA showed that the high-risk score group had a higher infiltrating proportion of CD4+ T cells, MDSCs, mast cells, Tregs and a lower infiltrating proportion of CD8+ T cells (**Figures 8G, H**). Additionally, we investigated the correlation between risk populations and immune checkpoint (ICP) expression. **Figures 8I** showed that expression of ICP molecules including CD86, VTCN1, NRP1, CD276, and PDCD1LG2 was significantly increased in high-risk patients, suggesting that high-risk patients may benefit more from treatment with these PCI inhibitors.

### 3.7 Assessment of tumor mutation burden and genetic mutation landscape in distinct risk groups

**Figure 9:**
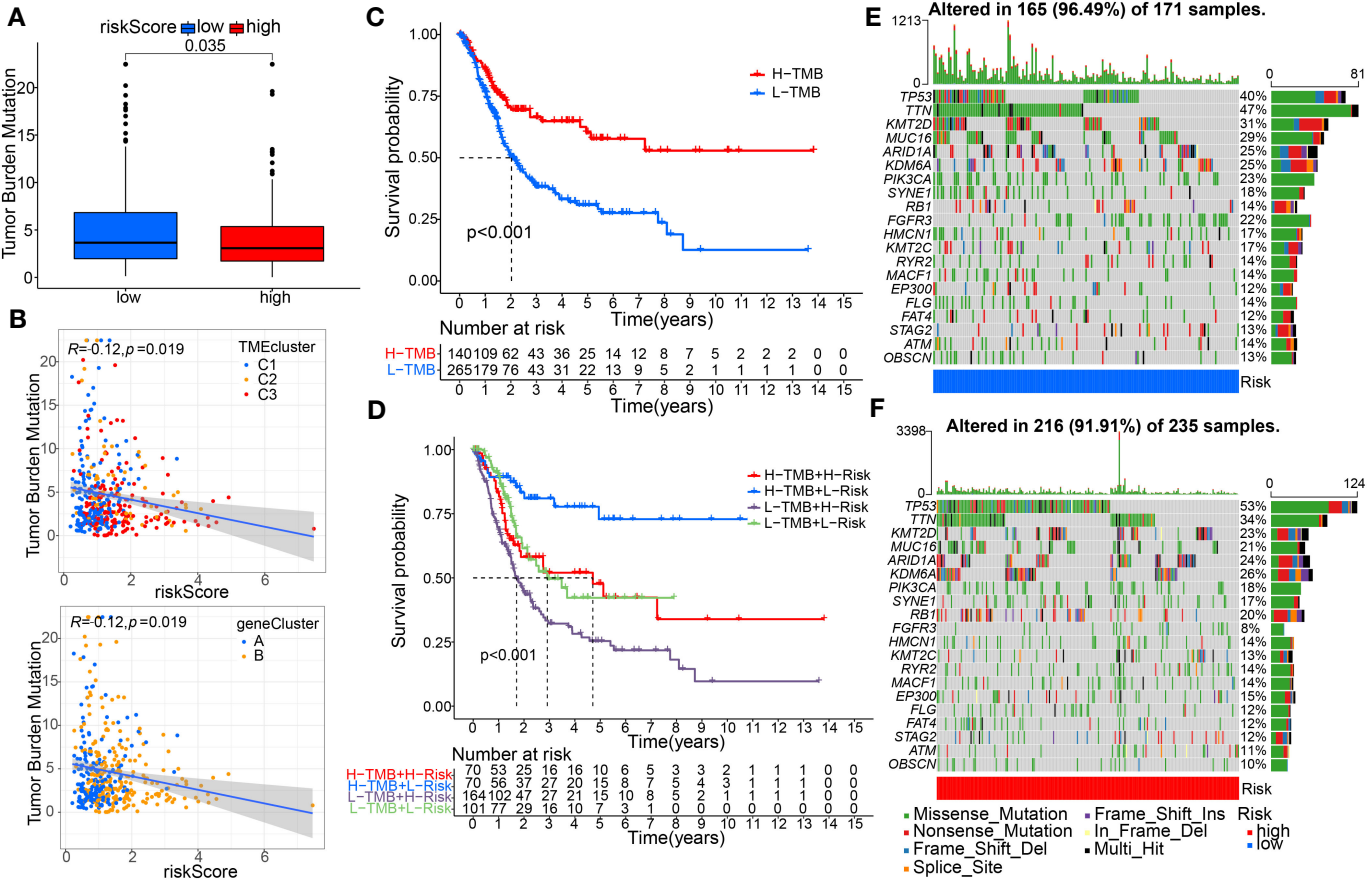
Evaluation of tumor mutation burden (TMB) and Genetic Mutation Landscape between the high- and low-risk groups. **(A)** The TMB difference analysis between the two groups. **(B)** Spearman correlation analysis with distinguishing clusters among risk score and TMB was performed. **(C)** Survival analysis of the OS between the low- and high-TMB groups. **(D)** K–M analysis among four patient groups stratified by both TMB and risk score. **(E, F)** The waterfall plot of somatic mutation features was distinguished with high and low risk scores.

Our results showed that the low-risk group had a higher tumor mutational burden (TMB) than the high-risk group. Spearman’s correlation analysis showed a negative correlation between risk score and TMB (**Figures 9A, B**). The KM curve showed that patients with high TMB had a better prognosis than those with low TMB (**Figure 9C**). Next, we integrated TMB and risk scores to examine their potential impact on BC patient outcomes. **Figure 9D** shows that both high-TMB and low-risk patients may benefit more from immunotherapy. Furthermore, we examined differences in the distribution of detected somatic mutation signatures between high-risk and low-risk groups in the TCGA-BC dataset. The mutation rates of TP53, TTN, KMT2D, MUC16, ARID1A and KDM6A genes in both groups of BC patients were higher than 20% (**Figures 9E, F**).

### 3.8 The role of the model in the modulation of the tumor immune microenvironment and immunotherapy

**Figure 10:**
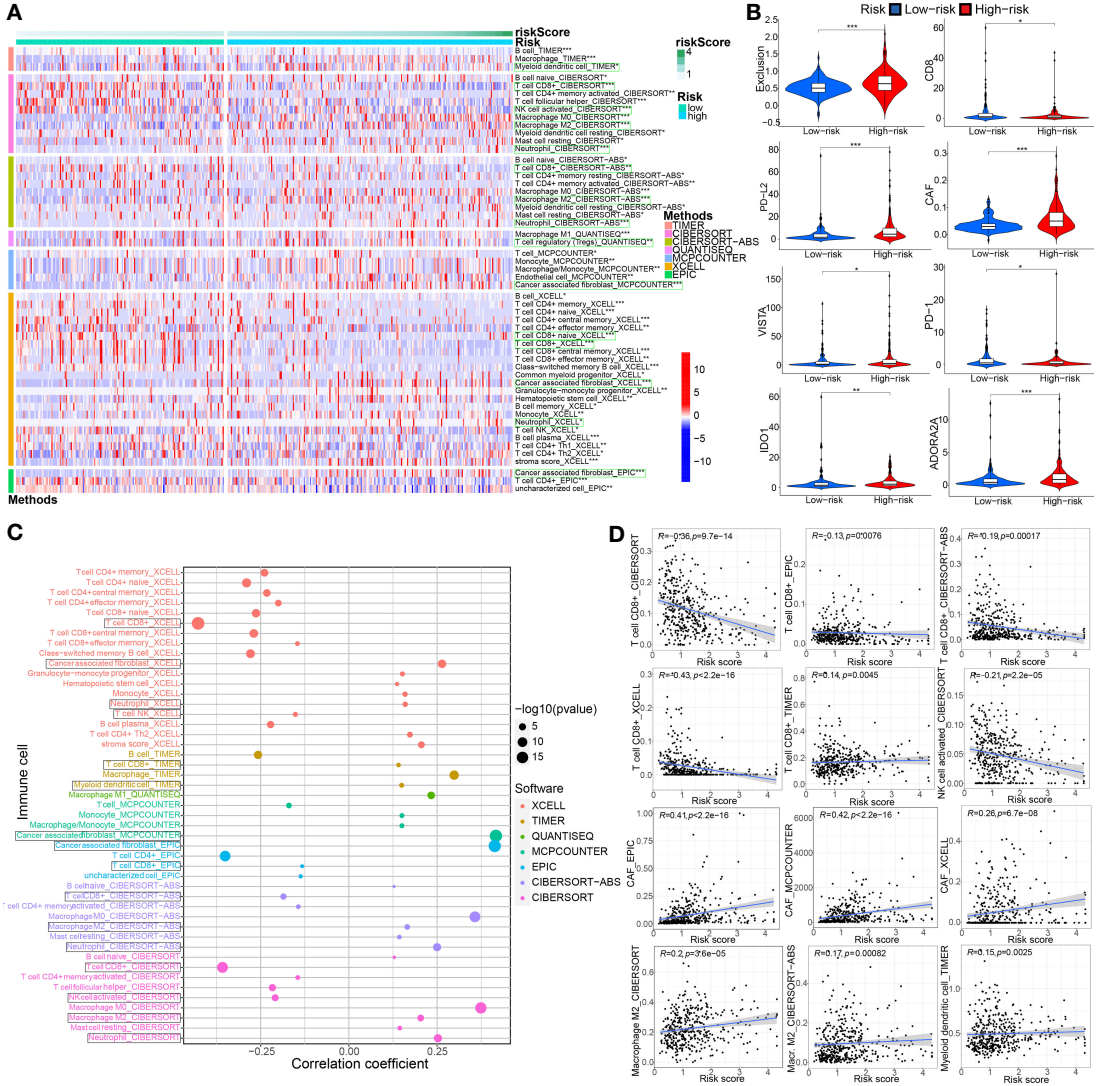
Associations between TME-associated gene signature and immune-cell infiltration, immune checkpoint immunotherapies. **(A)** There were distinct differences in 54 immune cells infiltration in the high- vs. low-risk groups. **(B)** The TIDE algorithm analysis and the expression of partial immune checkpoint genes was employed to predict the immune response to immune checkpoint therapy in BC patients based on riskscore. **(C, D)** Correlation analysis between infiltrating immune cells abundance from 7 immune-infiltration algorithm and the riskscore. *P< 0.05, **P< 0.01 and ***P< 0.001.

**Figure 11:**
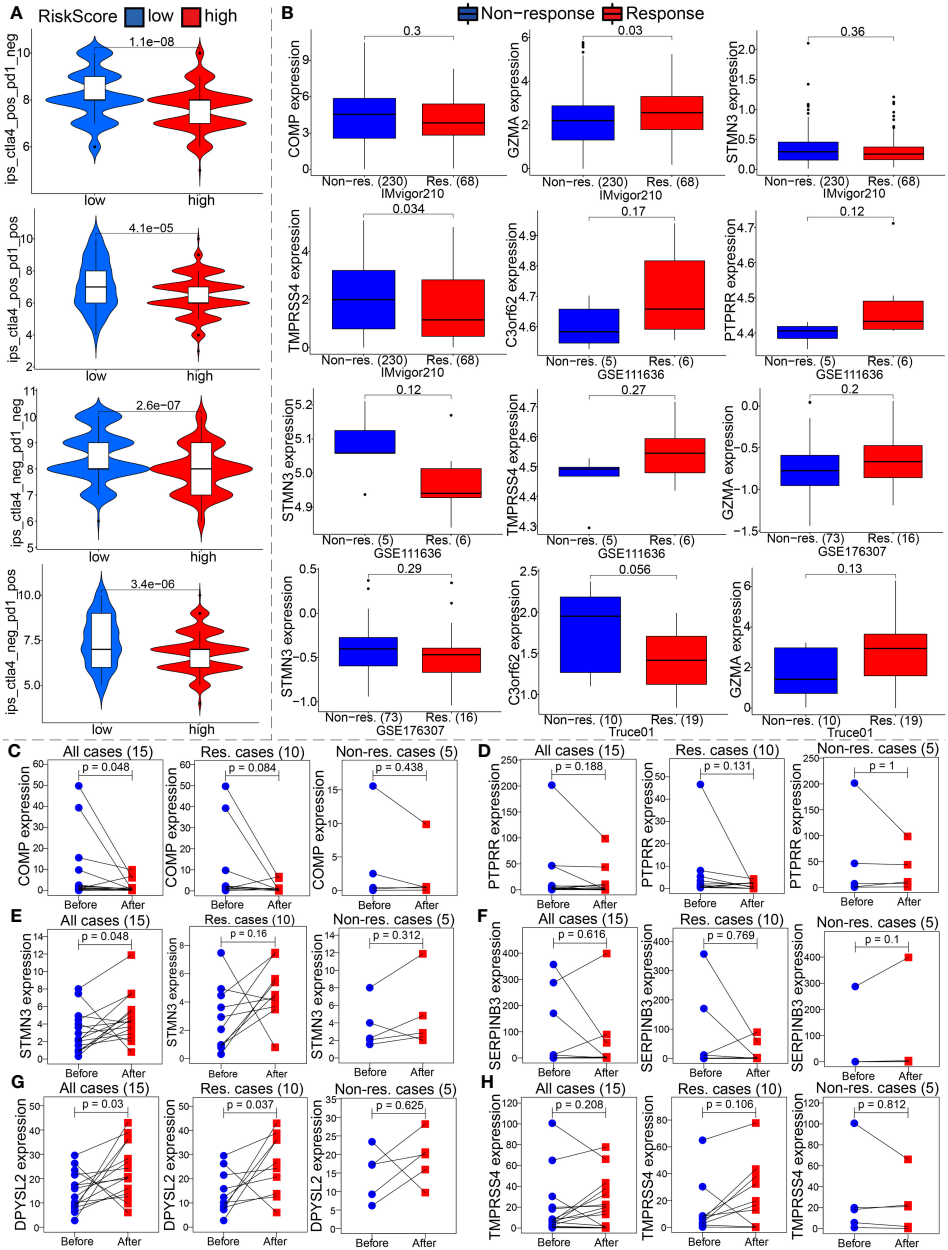
The model is an critical indicator of immunotherapeutic effect. **(A)** We evaluated the immunotherapy response by the immunophenoscore (IPS). **(B)** Differential expression analysis of partial model genes was conducted based on IMvigor210, GSE111636, GSE176307 and our own mRNA sequencing. **(C–H)** The differences in the partial model genes expression before and after immunotherapy were compared by paired Wilcox test in our TRUCE-01 data.

The extent of immune cell infiltration into the TME affects tumor development, disease course, and therapeutic efficacy, especially with immunotherapy. According to the 7 analysis of the immune infiltration algorithm (TIMER, CIBERSORT, CIBERSORT-ABS, QUANTISEQ, MCPCOUNTER, XCELL and EPIC), the heat map (Wilcoxon test, P<0.05) of 54 immune cells (sigDIC) was significantly different between the high-risk group and the low-risk group, as shown in **Figure 10A**. To forecast response to immunotherapy in two risk groups, TIDE algorithm analysis and partial immune checkpoint gene expression were used to predict immune checkpoint therapy response from group risk scores recorded by TCGA predictions. Compared with the low-risk group, the high-risk group showed higher rejection and expression levels of immune checkpoint molecules (**Figure 10B**). These diverse immune cells include CD8+ T cells, cancer-associated fibroblasts, M2 macrophages, neutrophils, and myeloid dendritic cells and T cell regulatory (Tregs), etc. Meantime, we performed Spearman correlation analysis (P< 0.01) on risk assessment and immune infiltrating cells (**Figures 10C, D**). In addition, to further evaluate the immune response in BC patients, we also calculated IPS. Our results showed that the low-risk group had a higher IPS score, suggesting that low-risk patients may be more sensitive to immunotherapy (**Figure 11A**).

To confirm our suspicions, we analyzed the relationship between the model and immunotherapy response in four immunotherapy datasets, namely IMvigor210, GSE111636, GSE176307 and Truce01. We discovered that GZMA expression levels were positively correlated with objective response to anti-PD-1/PD-L1 therapy in the Imvigor210 and Truce01 cohorts (**Figure 11B**). On the contrary, we noted the expression level of STMN3 was positively associated with resistance to cancer immunotherapy (anti-PD-1/PD-L1) in the Imvigor210 and GSE176307 (**Figure 11B**). Overall, these results indicated that the partial modeled genes expression can help predict the anti-PD-1/PD-L1 immunotherapy response. Apart from this, we also investigated the potential mechanism behind the expression of these model genes and immunotherapy response. Our own sequencing data found that the expression level of COMP/DPYSL2 in bladder cancer cases with response to tislelizumab combined with nab-paclitaxel therapy significantly decreased after treatment; however the expression level of TMPRSS4 increased after treatment in non-responsive cases (**Figures 11C–H**). Therefore, these results indicated that the molecular mechanism underlying these responses correlated significantly with model gene expression. In future work, we further investigated the molecular mechanism.

### 3.9 Drug sensitivity analysis of the model

Subsequently, to determine the performance of a prognostic model as a biomarker in the management of BC patients, we conducted a spearman correlation analysis between 142 drugs and 9 model genes based on the CellMiner database (**Supplementary Table S8**). **Supplementary Figure 6A** showed that GZMA could predict the sensitivity of Nelarabine, Dexamethasone Decadron, Fluphenazine, Arsenic trioxide, Fludarabine, and Cyclophosphamide; SERPINB3 could predict the sensitivity of Procarbazine, Olaparib, TESTOLACTONE, Calusterone, Simvastatin, VINORELBINE, and Dromostanolone Propionate; C3orf62 can forecast susceptibility to nelarabine, fludarabine, fluphenazine, and dasatinib. COMP forecast susceptibility to thiotepa, idarubicin, and triethyleneamine. In addition, we also analyzed the correlativity between susceptibility to commonly used chemotherapeutic drugs for bladder cancer and the expression of template genes (**Supplementary Figure 6B**, p< 0.05). We observed that the expression of TMPRSS4, and STMN3 is negatively correlated with the drug IC50 of Gemcitabine, Cisplatin, Paclitaxel, or Carboplatin. However, C3orf62, and COMP were positively correlated with the IC50 of Oxalitaxel, Cisplatin, or Vinblastine.

In addition, the IC50 values of four commonly used chemotherapeutic drugs (cisplatin, docetaxel, paclitaxel, and vinblastine) in the treatment of BC were analyzed using a pRRophetic algorithm. Sensitivity to paclitaxel, vinblastine, docetaxel, and doxorubicin was relatively higher in the high-risk group compared with sensitivity to these drugs in the low-risk group. The opposite was true for group C using mitomycin (P<0.01, Wilcox test; **Supplementary Figure 6C**). furthermore, we advance conducted a SPEARMAN correlation analysis between the risk score and the IC50 of the aforementioned chemotherapeutic drugs. As can be seen in **Supplementary Figure 6D**, the results of the correlation analysis (P<0.05, spearman correlation test) and the results of the Wilcox analysis of the above risk score group are unanimous. These results generally indicate that our prognostic model shows a strong correlation with drug sensitivity relevance.

### 3.10 Validation of partial modeled genes expression in bladder cancer; the biological function of SERPINB3 *in vitro*

**Figure 12:**
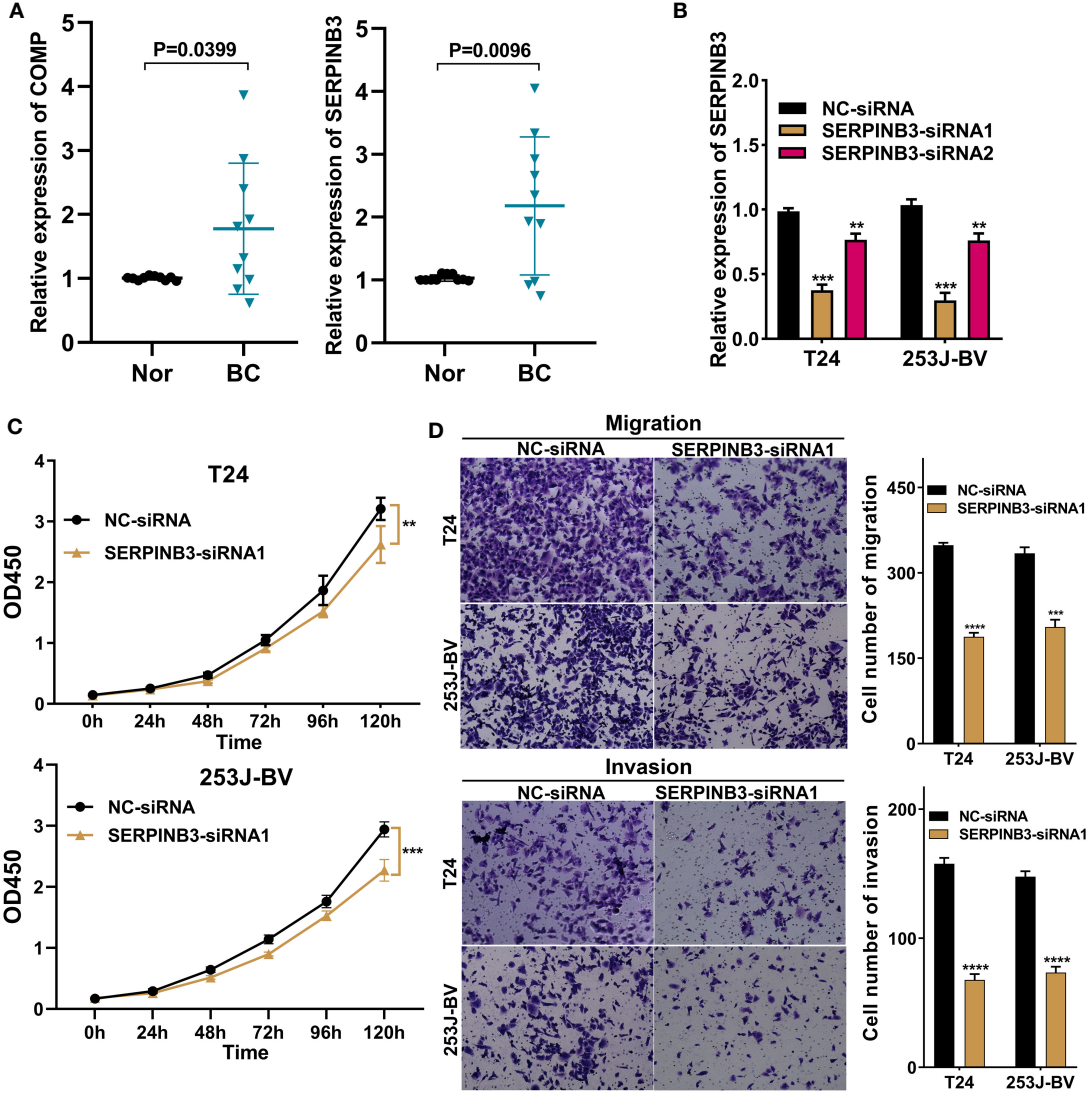
SERPINB3 gene played a tumor-promoting role in BC cells. **(A)** COMP and SERPINB3 genes were chosen to verify the differential expression results in 10 pairs of samples by qRT-PCR. **(B)** SERPINB3 small-interfering RNA (siRNA) transfection efficiency was assessed by qRT-PCR in T24 and 253J-BV cells. **(C)** CCK-8 assays were utilized to detect cell proliferation ability in T24 and 253J-BV cells transfected with NC-siRNA, and SERPINB3-siRNA1. **(D)** Migration and invasion were evaluated using a transwell assay without and with Matrigel, respectively. **P< 0.01, ***P< 0.001 and ****P< 0.0001.

First, we selected 10 pairs of BC tumor and cancer-adjacent normal tissues to verify COMP and SERPINB3 gene expression by real-time PCR (**Figure 12A**). Compared with expression in adjacent tissues, the expression of COMP and SERPINB3 is all upregulated in cancer tissues. Additionally, in light of the above observations, we explored the effects of SERPINB3 on the biological behavior of BC cells. We transiently transfected T24 and 253J-BV cells for 48 hours with siRNAs against SERPINB3 or control siRNAs. Transfection efficiency was verified by qRT-PCR (**Figure 12B**). The CCK-8 experiment results illustrated that the knockdown treatment of SERPINB3 suppressed significantly the proliferation ability of T24 and 253J-BV cell lines compared with NC (**Figure 12C**). Transwell experiments with or without Matrigel also showed that SERPINB3 knockdown greatly attenuated the migration and invasiveness of T24 and 253J-BV cell lines (**Figure 12D**). Taken together, these results suggest that SERPINB3 is essential for BC cell proliferation, migration and invasion.

## 4 Discussion

In this study, we systematically investigated molecular characteristics, clinical significance, and cancer immune interactions, and then constructed a novel prognostic model based on TME-related gene signatures in BC. Our findings indicate that this TME prognostic model could accurately predict prognosis and guide individualized treatment of BC patients.

At present, although treatment advances, the prognosis remains poor for both MIBC and advanced NMIBC, because of the molecular complexity and heterogeneity of BC (20). A large number of studies have shown that TME plays an important role in the occurrence, development and treatment of tumors. First, we used the TCGA-BC dataset to identify transcriptional changes and tmrg expression. The high mutational intensity of TMRGs in BC indicated that CNV gains and losses may contribute to cancer development or progression, which agrees with the findings of the previous study (21). Next, according to the expression similarity of prognostic DETMRGs, the merged BC samples were divided into three molecular clusters. High-level expression of C3 is associated with multiple clinicopathological parameters and poor prognosis. Concomitantly, there were significant differences in the accumulation of immune cell levels and functions among the three groups. We hypothesized that prognostic differences in TME clusters might be due to complex genetic heterogeneity of BC patients.

Afterwards, to advance survey the biological conduct behind each TME cluster, we then constructed two gene clusters based on 224 DEGs associated with the predictions. By LASSO and multiCox analysis, we constructed a novel prognostic model with nine genes consisting of C3orf62, DPYSL2, GZMA, SERPINB3, RHCG, PTPRR, STMN3, TMPRSS4, and COMP. Among these selected genes, increasing evidence has indicated that some of them may play different and crucial biological functions in the progression and prognosis of cancer. As shown in **Figure 5G**, patients in the high-risk group coinciding with high expression of DPYSL2, SERPINB3, RHCG, STMN3 and COMP have a worse prognosis. As discovered by Zou et al. (22), DPYSL2 upregulation correlated with tumor high-staging and poor prognosis in patients with BC, and promoted the malignant behavior of BC through enhancing aerobic glycolysis and EMT *in vitro* and *in vivo* experiments. Lauko et al. (23) suggested that SerpinB3 is necessary for cancer stem cells (CSCs) maintenance, tumor growth, and CSC pathway activation in glioblastoma, as well as inhibition of cathepsin L released from lysosomes leading to radiation resistance. Several previous studies have shown that SERPINB3 up-regulation caused by SDF-1/CXCR4/NF-kappaB pathway and HIF-2α-generated under hypoxic conditions separately facilitates the migration and invasion of gastric cancer (24) and hepatocellular carcinoma cells (25), exhibits superior spherogenic ability and invasion capacity of cholangiocarcinoma (26). Some studies have documented an important role of SERPINB3 in the modulation of programmed cell death by different mechanisms, both in inflammatory processes and in cancer (27). Chen et al. (28) found that RhCG is overexpressed in gastric cancer tissues versus normal tissues at mRNA and protein levels; and, its upregulation predicts poor survival and promotes migration and proliferation of gastric cancer via keeping intracellular alkaline. Similarly, STMN3, a microtubule destabilizing protein, is induced by both nicotine and EGF in an ID1 dependent fashion, as well as can facilitate the proliferation, invasion and migration of non-small cell lung cancer (29). As is well known, nicotine is the major ingredient of cigarette smoke, and smoking is the primary risk factor for bladder cancer (30), this might be the reason why STMN3 is highly expressed in the high-risk group of the model constructed by us. A pancancer multiomics analysis revealed that COMP may be a potential biomarker for pan-cancer diagnosis and prognosis, as well as its overexpression is linked with tumor immune evasion (31). Futhermore, COMP was a crucial CAFs-driven gene associated with the infiltration of M2 macrophages and acted as a promising predictive capabilities for prognosis and immunotherapy response in patients with colon cancer (32).

On the one hand, as shown in **Figure 5G**, C3orf62, GZMA, PTPRR and TMPRSS4 showed the higher expression level in the low-risk group, and predicted favorable clinical prognosis for patients with BC. The expression of the GZMA, secreted by natural killer (NK) cells and effector cytotoxic T cells, was recently treated as an indicator of the intratumoral immune cytolytic activity (33). Zhou et al. (34) have shown that GZMA from natural killer cells and cytotoxic T lymphocytes (CTL) can kill GSDMB-positive cells through inducing pyroptosis, thus promotes CTL-mediated tumor clearance in mice. Epigenetic silencing of PTPRR resulting from DNMT3B-mediated methylation activates MAPK signaling, promotes metastasis and serves as a biomarker of invasive cervical cancer. And, PTPRR functions as a tumor suppressor in ovarian cancer by dephosphorylating and inactivating β-catenin (35), and in colorectal cancer via inhibiting the Ras/ERK/c-Fos signaling pathway (36). In contrast to previously reported results for multiple other types of cancer, our study finds that high expression of TMPRSS4 predicts a better clinical outcome in bladder cancer, and this finding has been proven in previous studies (37). Based on existing reports, the functional mechanism of some genes in this model in BC is still unclear and requires follow-up research for further exploration.

Furthermore, it is worth mentioning that among molecular clusters and gene clusters, cluster 3 and gene cluster B, which had the worst clinical outcomes, had the largest TMEscores. KM analysis showed that patients with high TMEScore had poorer OS; time-dependent ROC verified its predictive robustness to 1-, 3-, and 5-year OS. Besides, the high-risk group was remarkedly relevant to worse clinicopathological features, such as higher age, advanced T-stage, and N-stage. The analysis by MultiCox showed that TMEscore was an independent factor predicting the survival outcome of BC patients. Next, to better predict the survival of BC patients, we further constructed an individualized prognostic prediction model with nomograms using risk scores combined with clinical characteristics. Together, these findings showed the prognostic robustness of the novel prognostic model based on TME gene signature in BC patients.

To explore the underlying mechanisms of the predictive model, we conducted GO and KEGG enrichment analyses between the high- and low-risk groups. The results showed that ECM and collagen binding-related biological processes and pathways may contribute to BC progression by TME-related gene signatures. It is well known that ECM is a critical and active component of the TME. Collagen is predominant component of ECM, which stimulate invasion and metastasis by promoting cancer cell epithelia-mesenchymal transition (EMT) or collective invasion of cancer cells (38).

Moreover, immune interactions between tumors and the TME play a key role in tumorigenesis and could serve as therapeutic targets for BC (39). The composing and frequency of immune cells in the TME influence tumor progression and the efficacy of immunotherapy (40). To further investigate the relationship between this TME-based signature and immune status, we quantified the accumulated level of immune cell infiltration between the two ssGSEA risk groups. Interestingly, CD8+ T cells were significantly higher in the low-risk group. It is now recognized that CD8+ T cells are the main effector cells in cell-mediated antitumor immunity, which kills tumor cells by releasing perforin (41). Instead, studies have demonstrated that immunosuppressive factors such as MDSCs, mast cells, Tregs evade surveillance and clearance of the immune system by different mechanisms (42). This is consistent with our results of abundant MDSCs, mast cells (MCs), and Tregs in high-risk group BC patients. At the same time, the enrichment of CD4+ T cells in high-risk group seems to contradict what is commonly believed that high immune infiltration of CD4+ T cells have better immune response. One review (43) concluded that CD4+ T cells in the TME have dual anti-tumor and pro-tumor effects. Besides, they may interact in combination with other types of immune cells, such as MDSCs, Tregs and tumor-associated Macrophages (TAMs), in shaping the cancer immune microenvironment. Oliveira et al. (44) suggested that CD4+ T cells can kill tumor cells by assisting CD8+ T function as helper cells or acting as CD4 cytotoxic T lymphocytes to kill tumors directly. Enrichment of CD4+ T cells associated with these two pathways in the tumor microenvironment generally leads to a better immune response and a better prognosis. However, some additional CD4+ T cells are immunosuppressive cells, which are involved in immune escape of tumor cells; thus, when the infiltration levels of these cells increased in the TME, in turn promoting tumor progression and contributing to poor prognosis. Myeloid cells, such as MDSCs TAMs, etc, have a dominantly immunosuppressive role (45); as well as targeting these cells might be an alternative and promising target for immunotherapy, and enhancing the efficacy of tumor immunotherapy (46, 47). A previous study showed that tumor-infiltrating mast cells colocalize with regulatory T cells, coincide with local reduction of MHC-I and CD8 T cells, and is associated with anti-PD-1 resistance, which can be reversed by c-kit inhibitor treatment. At present, some studies suggested that MCs possess enormous capabilities to shape the immune microenvironment (48, 49) and are becoming a new player in the field of cancer immunotherapy, depletion of these cells or downregulation of their functions in the TME can help break tumor resistance to anti-PD-1 therapy (50). Accordingly, we speculate that the TME prognostic model may affect BC survival outcomes by reshaping the tumor immune microenvironment, such as altering ECM, CD8+ T cell and these above-mentioned immunosuppressive cells, etc.

In addition, the findings from seven immune-infiltration algorithm analyses revealed a noteworthy positive correlation between the risk score and the presence of cancer-associated fibroblasts (CAFs), T cell regulatory (Tregs), macrophage M2, myeloid dendritic cell, and neutrophils. Conversely, the risk score demonstrated a significant negative association with the infiltration level of CD8+ T and NK-T cells from multiple algorithms (including CIBERSORT, CIBERSORT-ABS, EPIC, and XCELL). However, the risk score was positively correlated with CD8+ T cells based on TIMER algorithm. In a prior investigation, it was found that cancer-associated fibroblasts (CAFs), which represent the activated form of fibroblasts and are the predominant and diverse stromal cells in the tumor microenvironment (TME), play a critical role in the development, progression, chemoresistance, ECM remodeling, and response to anti-PD1/PD-L1 immunotherapy in multiple cancer types (51–53). One study demonstrated that one the one hand, the impact of CAFs on immune cell function is mediated through the secretion of diverse cytokines and products. On the other hand, as an integral constituent of the tumor stroma, CAFs contribute to the formation of a permeability barrier through stromal remodeling, consequently diminishing the efficacy of drug-based therapeutic interventions (54). A recent review suggested that the presence of CAFs high heterogeneity and their complex interaction with TME, influences responsiveness of anti-PD-1/PD-L1 immunotherapy (53). For instance, It can promote Treg, neutrophils recruitment, migration and differentiation, remodel ECM, and exclude CD8 T cells; as well as it can contribute to monocyte recruitment, induce TAMs to M2 phenotypic differentiation, up-regulate the expression level of PD-L1 on the surface of the TAMs, and impair its phagocytosis and effector T cell function (55, 56).

One systematic review indicated also that Tregs have the immune suppressor function and affect the immune response of monoclonal antibody-based immune checkpoint inhibitors through a variety of pathways, such as down-regulating of CD80 and CD86 co-stimulatory molecules, enhancing interaction of PD-L1/PD-1 and CTLA4/CD80, as well as promoting secretion of cytokines, including IL-10, TGF-b, and IL-35, and production of adenosine to regulate APC activity (57). MDSCs are a population of immature myeloid cells that suppress adaptive immune function, utilizing a variety of pathways, such as arginase, IL-10, IL-4, iNOS, reactive oxygen species, induction of other regulatory cell populations such as regulatory T cells, and their potent suppressive activities against effector lymphocytes (47, 58, 59) Additionally, previous evidence has shown that a high infiltration of M2 macrophages and a low presence of CD8 T cells in the high-risk group of BC are associated with a poor response to immunotherapy (60). Through single-cell RNA sequencing, Chen et al. also demonstrated that monocyte/macrophages polarization toward M2 phenotype, LAMP3 + DC subgroup recruiting regulatory T cells, and inflammatory cancer-associated fibroblasts (iCAFs) in the tumor region, are all potentially implicated in the formation of an immune-suppressive TME and tumor progression, which are strongly correlated with poor prognosis of BC patients (61).

Recently, immune checkpoint inhibitor therapy has emerged as a promising treatment option for BC (62). Our research showed that several ICP molecules as potential targets for immunotherapy exhibit higher expression in the high-risk group, such as CD86, VTCN1, NRP1, CD276, and PDCD1LG2. Besides, The TIDE model, developed by Jiang et al. (63), aims to predict the probability of immunotherapy responsiveness by modeling the mechanisms of tumor immune evasion. Notably, our study revealed that distinct immune characteristics, differential expression of immune checkpoint genes (including PD-1, PD-L2, VISTA, IDO1, and ADORA2A), and varying immunotherapeutic responses between high- and low-risk groups. Consequently, the integrative analysis suggests that the subgroup of BC patients classified as high-risk may experience enhanced immunotherapy efficacy. Of them, VISTA, a transmembrane protein of type I, belongs to the B7 family and plays a crucial role in maintaining the quiescence of T cells and myeloid cells, and is a promising target for combination cancer immunotherapy (64). In addition to its role as a ligand expressed on antigen-presenting cells, VISTA also functions as a receptor on T cells. Previous research has predominantly focused on elucidating the suppressive impact of VISTA on the immune system and investigating the potential of VISTA-deficiency or anti-VISTA treatment in enhancing immune response (65, 66). IDO1 is a rate-limiting metabolic enzyme that converts the essential amino acid tryptophan (Trp) into downstream catabolites known as kynurenines, creates an immunosuppressive environment, and is suggested as having an important role in contributing to resistance to immunotherapy (67, 68). In addition, increasing evidence confirms that TMB is a predictive biomarker for tumor progression-free survival and immune response (69). Higher BMR has been shown to have a better prognosis in BC patients, which is consistent with our findings (70). The high-risk and low-risk BMR groups had significantly better survival than the other groups, suggesting that TMB combined with TMEscore may be a prognostic biomarker for BC. Taken together, our data indicate that poor prognosis of high-risk patients might be correlated with immunosuppressive TME of BC.

There is evidence to suggest that BC patients treated with neoadjuvant chemotherapy, immunotherapy and targeted therapy can reduce tumor progression and improve outcomes in BC patients (71). Predicting responses to immunotherapy requires specific biomarkers. Thus, we evaluated ICIs response by creating IPS signatures, and discovered that BC patients with lower TMEscore showed positive responses to anti-PD1 and anti-CTLA-4 treatments. TIDE was developed by Jiang et al. (63) based on the modeling of tumor immune evasion mechanism to predict the response to immunotherapy. Moreover, to overcome drug resistance and improve clinical outcomes with BC patients, we identified the potential drugs targeting TME prognostic model or model-related genes. Nevertheless, there are some limitations to the drug sensitivity findings of this model that were merely analyzed using two public databases, that is Cellminer and Cancer Genome Project (CGP). These theoretical predictions need to be taken with caution and additional pre-clinical validation should be conducted. Of note, we also found that SERPINB3 gene can be used as a tumor regulator through qRT-PCR validation, which can provide diagnosis and prognosis prediction for future biomarkers in BC patients.

Several constraints are to be considered in our study. First, our study builds on a retrospective study of public datasets, and inherent selection bias may affect their robustness. Further prospective studies are required to validate the clinical value of this TME-based molecular signature. In addition, in order to reveal the underlying molecular mechanism of BC tumorigenesis, complementary experimental studies *in vivo* and *in vitro* are needed, confirming our findings.

In summary, we summarized the regulatory genes associated with the clinical significance and prognostic role of TME, and then established a new prognostic model based on nine TMRGs in BC. This prognostic model can accurately and steadily predict the survival of BC patients and guide the individualized treatment of patients. We further observed that changes in immune cell infiltration in the ECM and TME may be underlying mechanisms for BC development. The results of this study provide a worthful fundament for further research on the prognosis and individualized treatment of BC patients.

## Data availability statement

The datasets presented in this study can be found in online repositories. The names of the repository/repositories and accession number(s) can be found in the article/**Supplementary Material**.

## Ethics statement

Ethical approval was not required for the studies on humans in accordance with the local legislation and institutional requirements because only commercially available established cell lines were used.

## Author contributions

HH, ZF and CS designed this study; CS, WC, and JH wrote the manuscript; ZZ, XL, and SY screened the database and collected the data; CS performed the bioinformatic analysis; HH, YW and CS revised the manuscript; DW, and FW provided critical comments; All authors contributed to the article and approved the submitted version.

## Supplementary material

Supplementary Figure 1 Screening and genetic mutation landscape of prognostic DE-TMGs. **(A)** Venn diagram of the intersection with DE-TMGs and prognostic TMGs. **(B, C)** Expression heatmap and forest plot for prognostic DE-TMGs between BC and normal tissues. **(D)** The PPI network among prognostic DETMGs. **(E)** Genetic mutation frequency and types of prognostic DE-TMGs. **(F, G)** Frequencies of the copy number variation (CNV) gain and loss among the top 20 genes in gain and loss-CNV groups. **(H)** The location of CNV alteration of prognostic DETMGs on 23 chromosomes. (p< 0.05 *; p< 0.01 **; p< 0.001 ***).

Supplementary Figure 2
**(A)** Top30 differential KEGG pathways between C1 and C2 by GSVA method. **(B)** Top30 differential KEGG pathways in C3 vs. C2 by GSVA. **(C)** Intersection of differential KEGG pathways in comparison between C1 and C2, C1 and C3, C2 and C3.

Supplementary Figure 3 The correlation analysis of risk score and clinicopathological characteristics in BC, including Age, Sex, Grade, T-stage, N-stage, and Survival status, by Chi-square test and Wilcox nonparametric test **(A-F)**.

Supplementary Figure 4 External verification based on GSE32894 and GSE48075 cohorts. **(A-C)** KM, ROC and calibration curves were plotted from GSE32894 cohort. **(D-G)** The associations between the model and clinicopathological features, including stage_T, molecular subtype, grade, and stage N, according to GSE32894 dataset. **(H-J)** KM, ROC and calibration curves were presented using the GSE48075 cohort.

Supplementary Figure 5 Based on different clinical traits, the subgroup survival analysis was performed between high and low risk group in BC patients. **(A, B)** Age (age ≤ 65 and age > 60 years old). **(C, D)** Gender (male and female). **(E, F)** T-stage (T1-2 and T3-4). **(G, H)** Grade (Low and High).

Supplementary Figure 6 Relationship between the modeled genes expression levels and small molecular chemotherapy drug sensitivity. **(A)** Top 25 drugs significantly associated with partial model genes (P<0.01) were obtained. **(B)** The significant correlation between commonly used chemotherapeutic drugs for BC and the expression of model genes (P<0.05). **(C)** Wilcox group analysis and **(D)** spearman correlation analysis indicated that the TME-associated gene model is robust to drug sensitivity of Docetaxel, Doxorubicin, Mitomycin C, Paclitaxel and Vinblastine.
