# Supplementary figures and images for: Identification and validation of a dysregulated TME-related gene signature for predicting prognosis, and immunological properties in bladder cancer
Source: Front Immunol. 2023 Oct 27;14:1213947. doi: 10.3389/fimmu.2023.1213947 (PMC10641729; doi:10.3389/fimmu.2023.1213947)

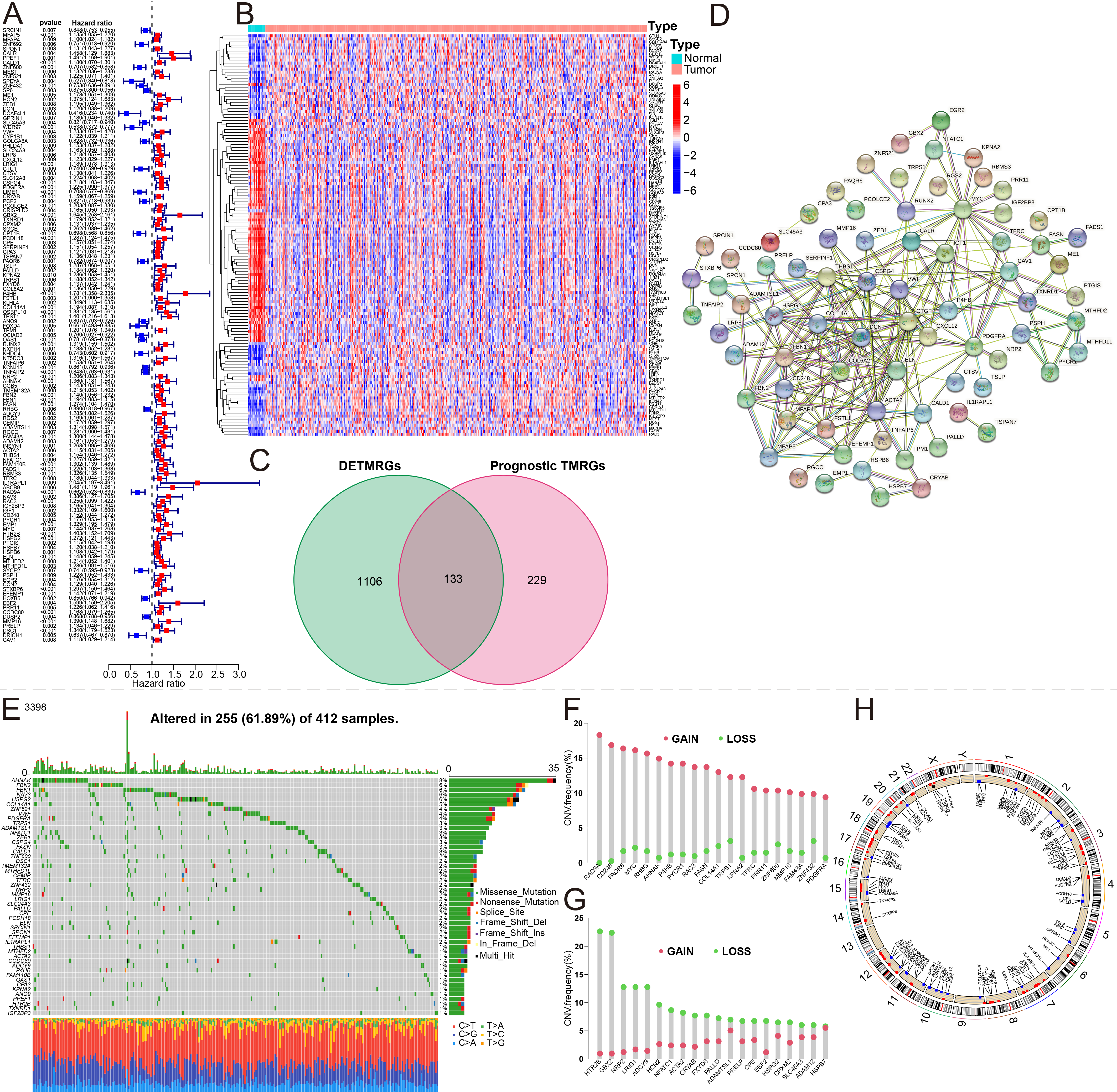

Supplement: Supplementary Figure 1 — Screening and genetic mutation landscape of prognostic DE-TMGs. (A) Venn diagram of the intersection with DE-TMGs and prognostic TMGs. (B, C) Expression heatmap and forest plot for prognostic DE-TMGs between BC and normal tissues. (D) The PPI network among prognostic DETMGs. (E) Genetic mutation frequency and types of prognostic DE-TMGs. (F, G) Frequencies of the copy number variation (CNV) gain and loss among the top 20 genes in gain and loss-CNV groups. (H) The location of CNV alteration of prognostic DETMGs on 23 chromosomes. (p< 0.05 *; p< 0.01 **; p< 0.001 ***). [file Image_1.tif]

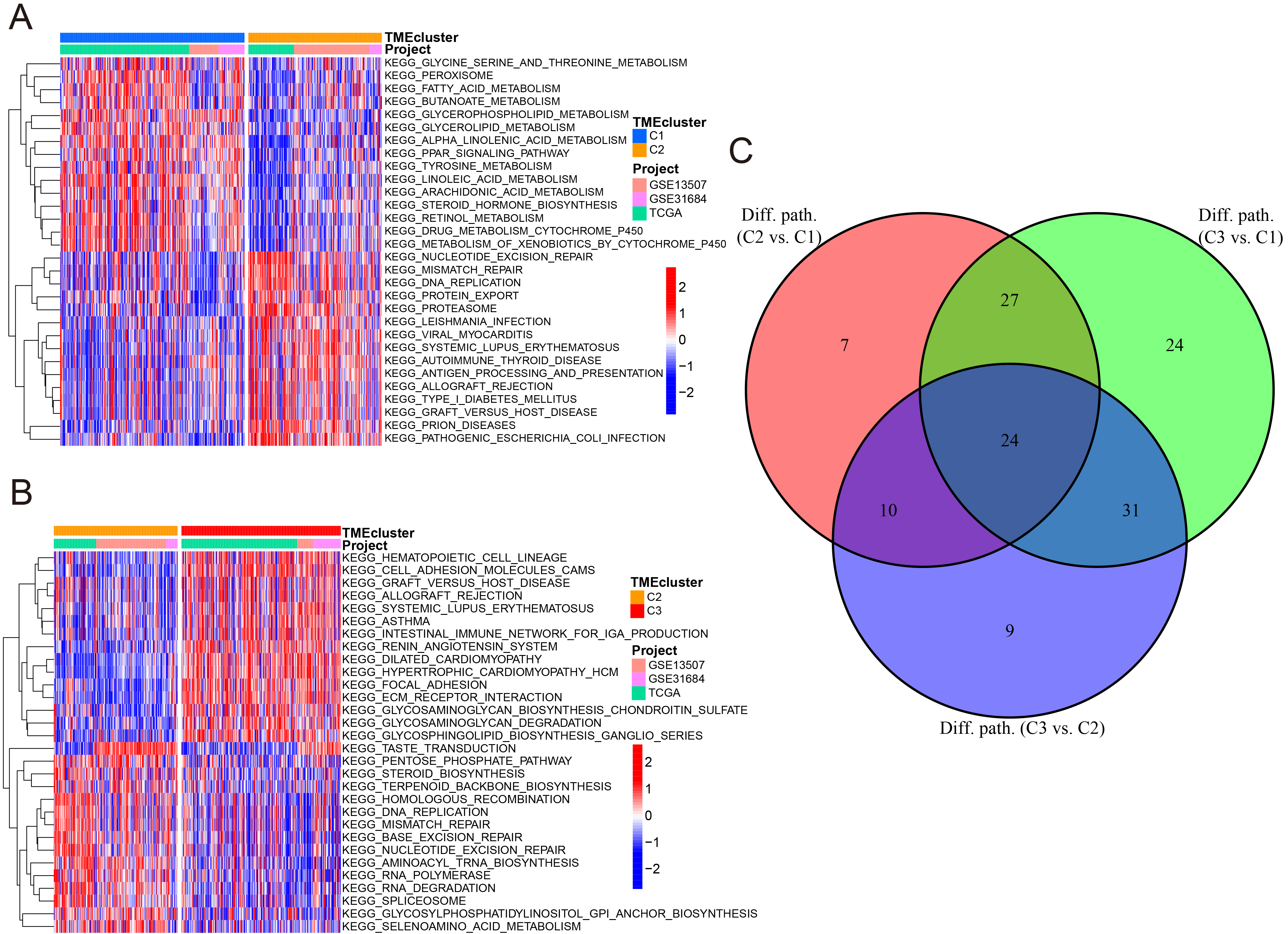

Supplement: Supplementary Figure 2 — (A) Top30 differential KEGG pathways between C1 and C2 by GSVA method. (B) Top30 differential KEGG pathways in C3 vs. C2 by GSVA. (C) Intersection of differential KEGG pathways in comparison between C1 and C2, C1 and C3, C2 and C3. [file Image_2.tif]

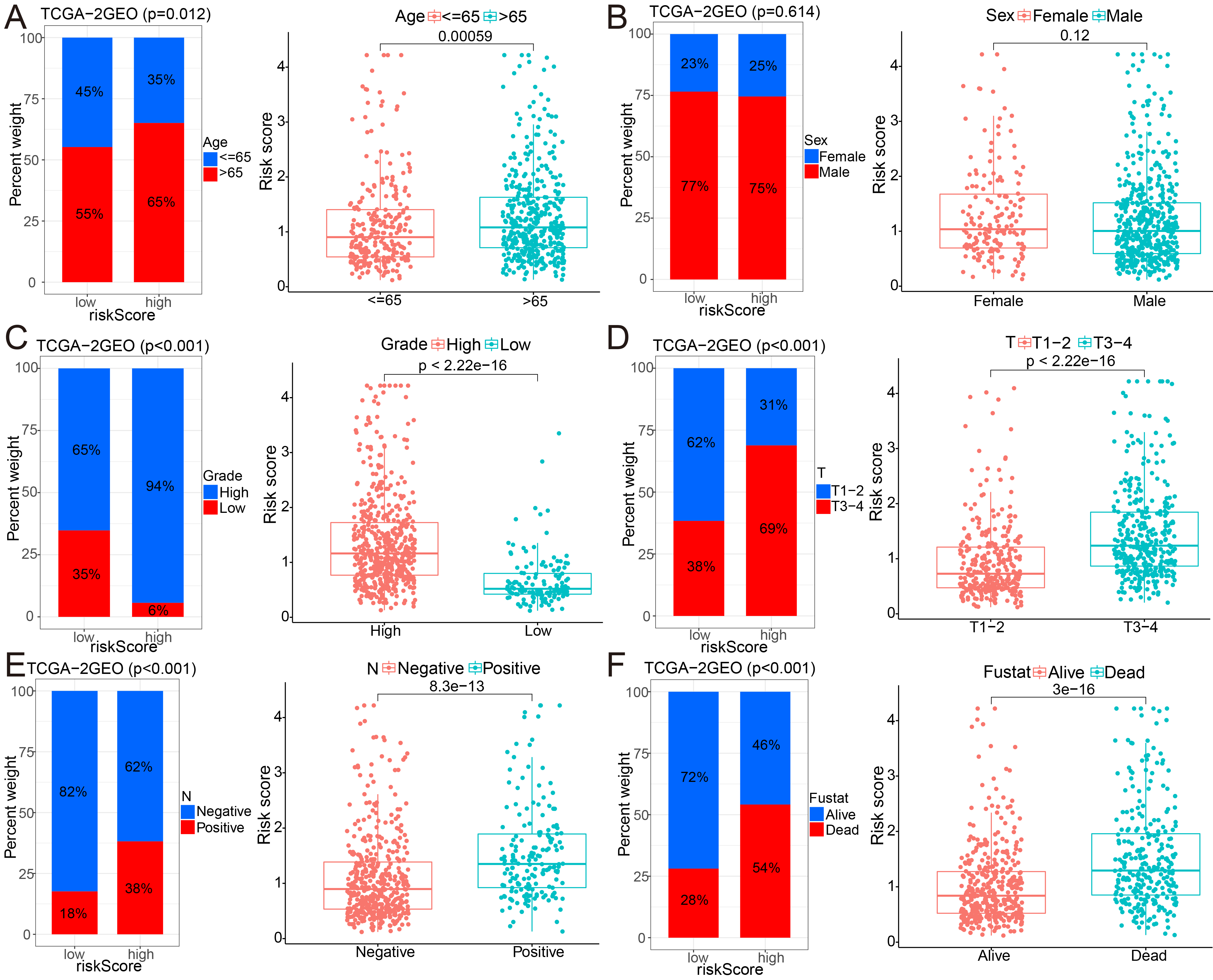

Supplement: Supplementary Figure 3 — The correlation analysis of risk score and clinicopathological characteristics in BC, including Age, Sex, Grade, T-stage, N-stage, and Survival status, by Chi-square test and Wilcox nonparametric test (A-F). [file Image_3.tif]

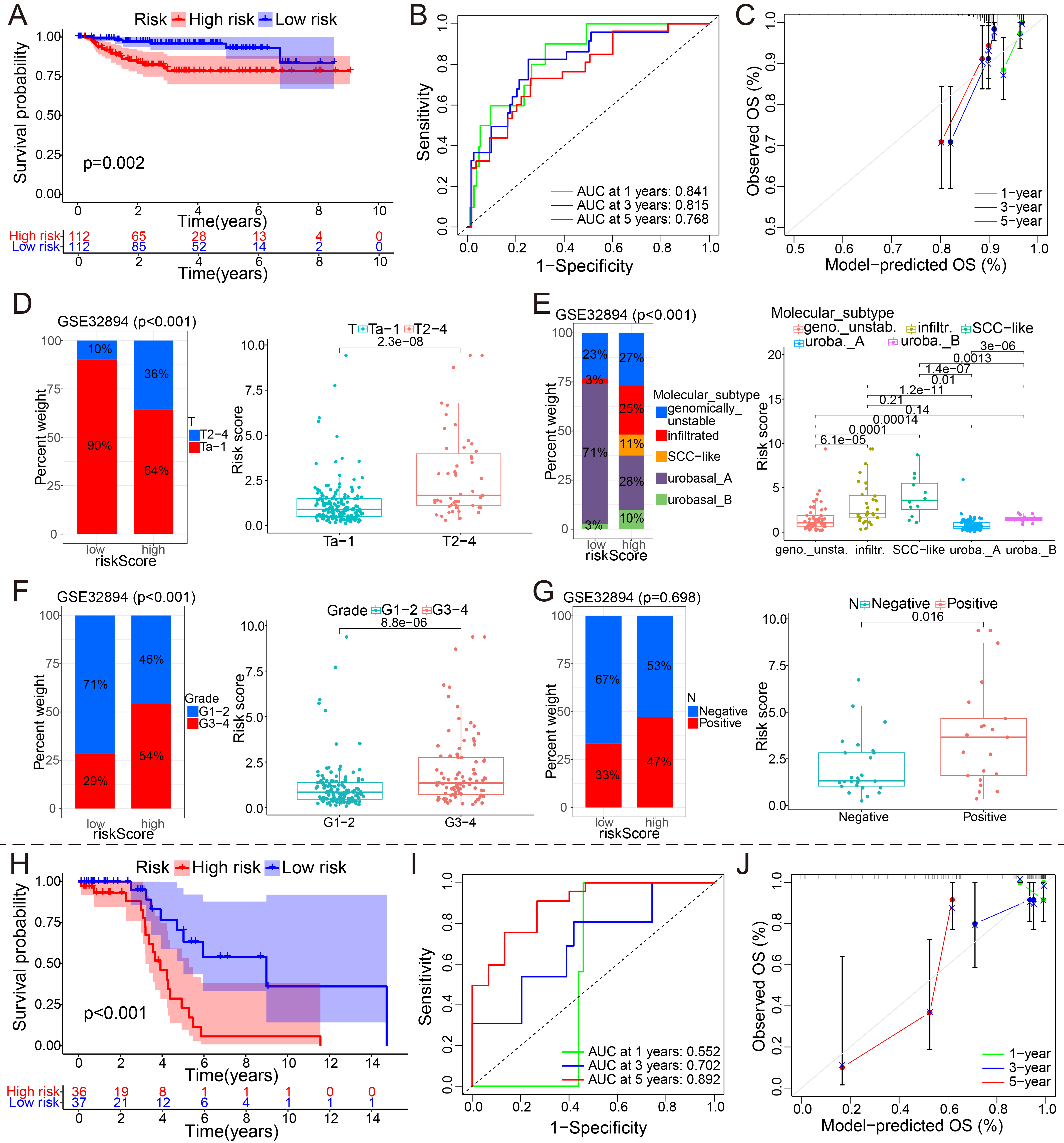

Supplement: Supplementary Figure 4 — External verification based on GSE32894 and GSE48075 cohorts. (A-C) KM, ROC and calibration curves were plotted from GSE32894 cohort. (D-G) The associations between the model and clinicopathological features, including stage_T, molecular subtype, grade, and stage N, according to GSE32894 dataset. (H-J) KM, ROC and calibration curves were presented using the GSE48075 cohort. [file Image_4.tif]

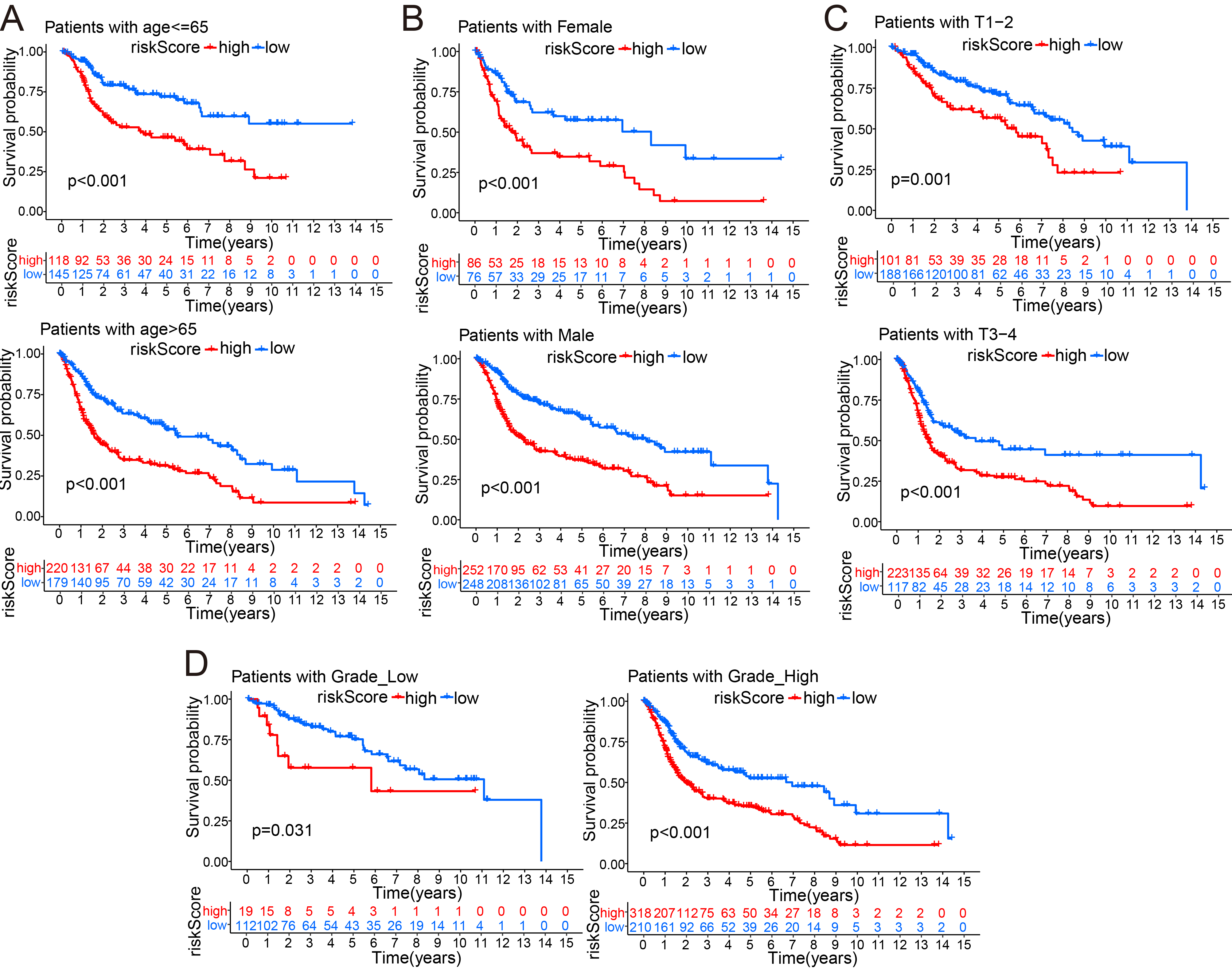

Supplement: Supplementary Figure 5 — Based on different clinical traits, the subgroup survival analysis was performed between high and low risk group in BC patients. (A, B) Age (age ≤ 65 and age > 60 years old). (C, D) Gender (male and female). (E, F) T-stage (T1-2 and T3-4). (G, H) Grade (Low and High). [file Image_5.tif]

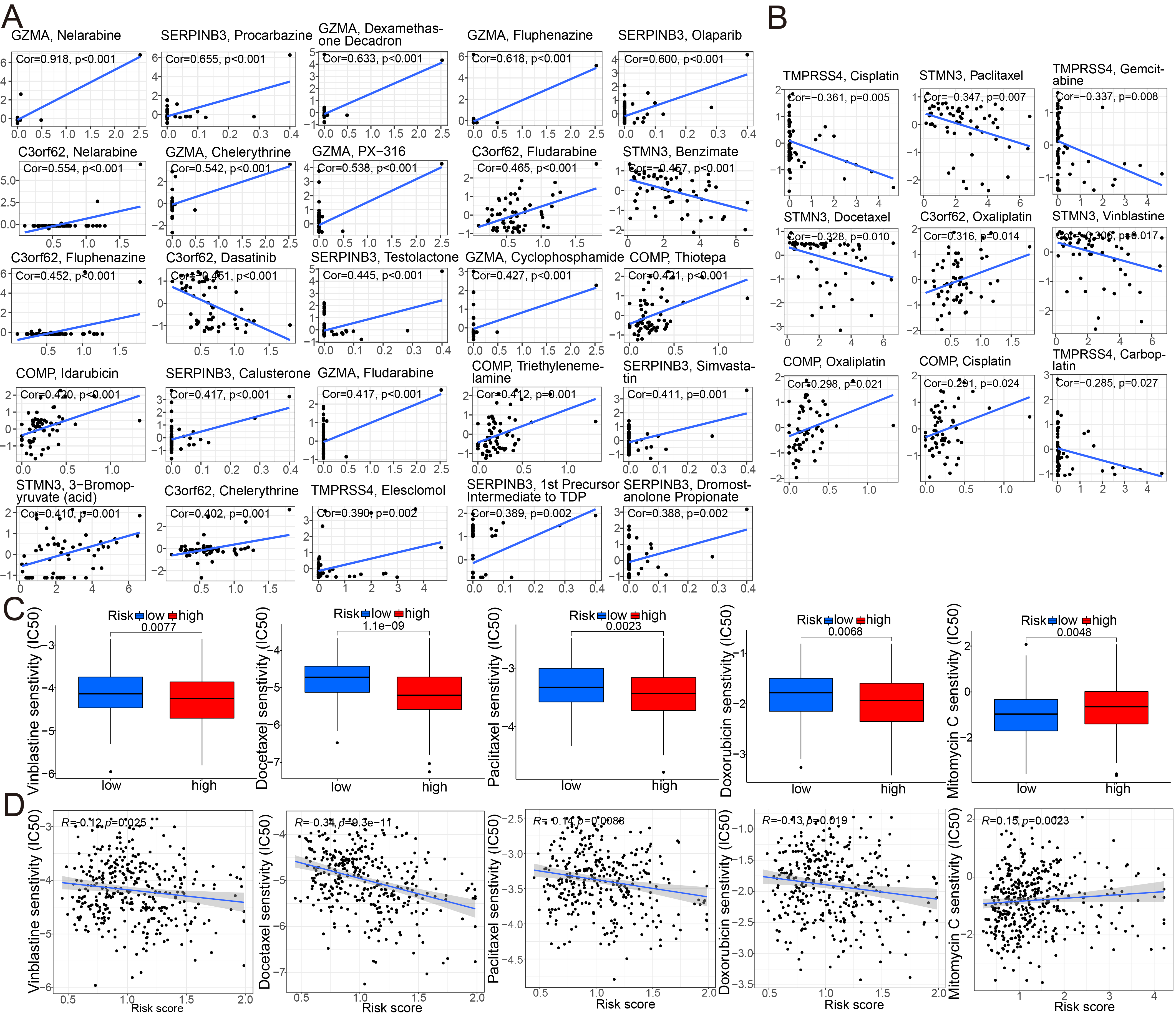

Supplement: Supplementary Figure 6 — Relationship between the modeled genes expression levels and small molecular chemotherapy drug sensitivity. (A) Top 25 drugs significantly associated with partial model genes (P<0.01) were obtained. (B) The significant correlation between commonly used chemotherapeutic drugs for BC and the expression of model genes (P<0.05). (C) Wilcox group analysis and (D) spearman correlation analysis indicated that the TME-associated gene model is robust to drug sensitivity of Docetaxel, Doxorubicin, Mitomycin C, Paclitaxel and Vinblastine. [file Image_6.tif]
